# Multiscale mutation clustering algorithm identifies pan-cancer mutational clusters associated with pathway-level changes in gene expression

**DOI:** 10.1371/journal.pcbi.1005347

**Published:** 2017-02-07

**Authors:** William Poole, Kalle Leinonen, Ilya Shmulevich, Theo A. Knijnenburg, Brady Bernard

**Affiliations:** Institute for Systems Biology, Seattle, WA, United States of America; University of Maryland Baltimore County, UNITED STATES

## Abstract

Cancer researchers have long recognized that somatic mutations are not uniformly distributed within genes. However, most approaches for identifying cancer mutations focus on either the entire-gene or single amino-acid level. We have bridged these two methodologies with a multiscale mutation clustering algorithm that identifies variable length mutation clusters in cancer genes. We ran our algorithm on 539 genes using the combined mutation data in 23 cancer types from The Cancer Genome Atlas (TCGA) and identified 1295 mutation clusters. The resulting mutation clusters cover a wide range of scales and often overlap with many kinds of protein features including structured domains, phosphorylation sites, and known single nucleotide variants. We statistically associated these multiscale clusters with gene expression and drug response data to illuminate the functional and clinical consequences of mutations in our clusters. Interestingly, we find multiple clusters within individual genes that have differential functional associations: these include *PTEN*, *FUBP1*, and *CDH1*. This methodology has potential implications in identifying protein regions for drug targets, understanding the biological underpinnings of cancer, and personalizing cancer treatments. Toward this end, we have made the mutation clusters and the clustering algorithm available to the public. Clusters and pathway associations can be interactively browsed at m2c.systemsbiology.net. The multiscale mutation clustering algorithm is available at https://github.com/IlyaLab/M2C.

## Author summary

Identifying driver mutations in cancer has been a major challenge in cancer research, with the ultimate goal of understanding the detailed molecular origins of cancer and providing genetically personalized treatments. For decades, the cancer research community has known that mutations in certain genes—such as tumor suppressors like P53—can drive cancer. In some cases it is also clear that mutations within cancer genes are localized in a single amino—such as the V600E mutation in BRAF. With the existence of large multi-omic data sets including The Cancer Genome Atlas (TCGA), it is now possible to apply big data approaches towards both identifying mutation features of interest and understanding their functional consequences. We have bridged the gap between single amino acid mutations and the whole gene view by developing an algorithm that can identify variable length regions within cancer genes that which enriched for mutations. Furthermore, we have been able to integrate our multiscale mutation clusters with additional molecular data to gain insight into possible functional consequences of the clusters.

This is a *PLOS Computational Biology* Methods paper

## Introduction

Somatic mutations are amongst the most frequent genomic aberrations associated with cancer. Moreover, primary human cancer samples usually contain tens to hundreds of somatic mutations, depending on the tissue of origin [1]. The identification of mutations that alter the function of protein coding genes, and a molecular understanding of the ensuing consequences of such mutations, remains a significant challenge.

To date, millions of distinct somatic mutations have been observed in human cancers through genome wide characterization projects such as The Cancer Genome Atlas (TCGA) and International Cancer Genome Consortium (ICGC). Computational methods are particularly well suited for the assessment of somatic mutations at this scale in order to identify those with cancer-associated functional consequences. To this end, numerous mutation assessment methods have been developed based on a variety of underlying approaches and statistical models. For example, MuSiC [2], MutSig [1], and SomInaClust [3] rank cancer-associated genes based on somatic mutations observed across a cohort of samples and normalized by factors including mutation type propensity, gene length, and replication timing. In addition, methods including PolyPhen [4], SIFT [5], and CADD [6] utilize prior knowledge such as conservation and machine learning based on disease associated variants to predict functional mutation impact. Yet other methods including OncodriveCLUST [7] and CLUMPS [8], iPAC [9], and graphPAC [10] take a parameterized, data-driven approach to predict cancer-associated mutations based on spatial clustering of linear sequence, three-dimensional protein structure, and graphical representations thereof. Finally, enrichment of somatic mutations based on functional biological consequences such as protein-protein interaction interfaces [11] and deregulation of phosphorylation signaling [12] have been explored.

Importantly, functional mutations that occur within the coding sequence and are known to be associated with cancer do not occur at random positions. On the contrary, hotspots or clusters are frequently observed as recurrent missense mutations across a significant fraction of cancer samples. These sets of mutations are typically attributed to alterations in function at specific sites of the protein that give rise to a variety of cancer phenotypes. Oftentimes, these mutation clusters can be readily interpreted in the context of their protein structure and function; for example, mutations in the GTP binding pocket of *KRAS* that modulates intrinsic GTPase activity, lead to constitutive activation of *KRAS* and persistent stimulation of downstream signaling pathways [13,14]. Such mutation clusters need not be located within structural protein domains; for example, N-terminal mutations of beta-catenin (*CTNNB1*) affect protein phosphorylation sites and thereby abrogate ubiquitin-mediated proteasomal degradation [15–17]. These mutations then result in beta-catenin accumulating in the nucleus and continuously driving transcription of its target genes [18,19].

While these examples highlight readily identifiable and interpretable focal mutation clusters that lead to cancer-associated effects on protein function, such mutation consequences need not be restricted to dense clusters at just a few amino acid positions in the protein sequence. For example, tumor protein p53 (*TP53*) contains a combination of mutations that are recurrently located at specific amino acids that bind directly to DNA (e.g., R248Q, R273C) as well as more broadly distributed sets of mutations throughout the core DNA binding domain of TP53 that disrupt the folding and stability of the protein [20,21]. Additionally, sets of mutations do not only affect individual regions or domains of proteins; rather, functional mutations are observed in distinct clusters within different regions or domains of individual proteins (e.g., *PIK3CA*), indicating the possibility for differential functional consequences of such mutation clusters [22,23].

We have therefore developed a multiscale mutation clustering algorithm (M^2^C) that identifies variable length regions with high mutation density in cancer genes. We have applied our algorithm on hundreds of frequently mutated genes using the combined mutation data in over twenty tumor types from TCGA and identified over a thousand multiscale mutation clusters. We have statistically associated these multiscale clusters with gene expression from TCGA tumor samples and drug response data from cancer cell lines, illuminating the (differential) functional and associated therapeutic consequences of somatic mutations in cancer.

## Results and discussion

### Overview of the approach

**Fig 1:**
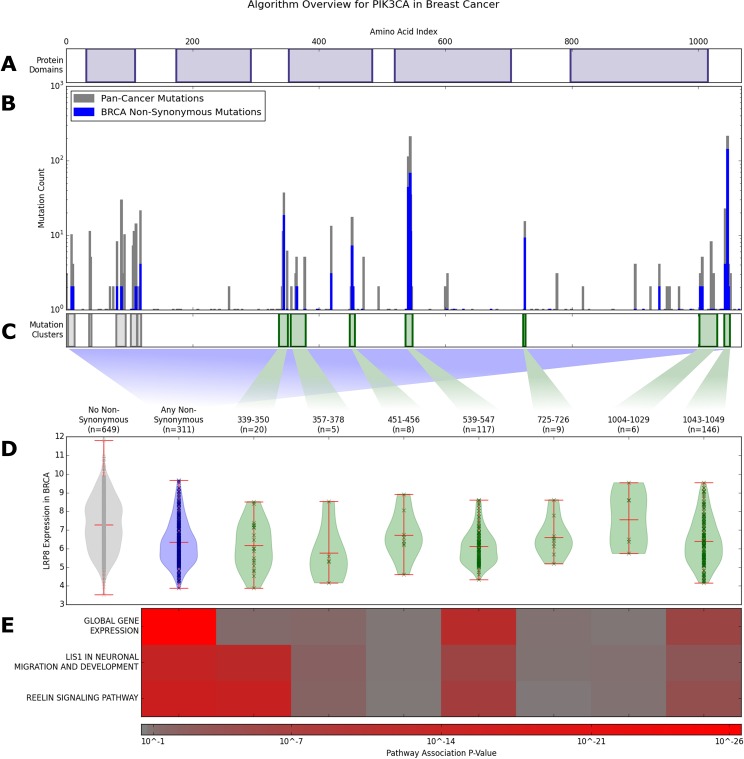
Method Illustration on PIK3CA in Breast Cancer: From top to bottom. **A)** Protein domains in PIK3CA. **B)** In gray: mutation histogram showing all mutations across the various cancer types; these data were used to generate the multiscale clusters. In blue: breast cancer mutation histogram showing all non-synonymous mutations; these data were used to assign mutation features to breast cancer tumor samples. **C)** Mutation clusters identified by the multiscale information-based clustering algorithm (M^2^C). Gray clusters have fewer than 5 mutations in breast cancer and are excluded from subsequent downstream analysis. Green clusters are assigned to breast cancer. **D)** LRP8 gene expression levels in breast cancer where the samples are grouped based on the mutation clusters. From left to right: “wild-type” (i.e., no non-synonymous mutations including tumors with no mutations at all), any non-synonymous mutation feature, and the seven mutation clusters assigned to breast cancer. **E)** Pathway association P-value heatmap showing differential pathway associations between clusters. L1S1 In Neuronal Migration and Development Pathway and Reelin Signaling Pathway do include LRP8.

We ran M^2^C on the combined mutation calls from 23 cancers (pan-cancer data set is described in T1 in S1 Tables) across 549 genes. Briefly, M^2^C works by smoothing the mutation density at many different amino acid length scales. Then, a mixture model is fit to each scale. Finally, these mixture models are merged together using a greedy algorithm that optimizes an information criterion. We refer to a cluster as an interval along the linear amino acid chain, e.g. *PIK3CA* 339–350. After identifying these clusters, we assigned them as binary features to individual tumor types for each of the 23 cancers. A cluster is assigned as positive (1) to a tumor sample if that sample contains at least one non-synonymous mutation within the cluster and negative (0) otherwise. This assignment allowed us to relate cluster features with gene expression data from 2194 genes in the TCGA dataset. We statistically combined these gene expression associations on the pathway level across 172 pathways linking mutation clusters to pathway-level gene expression changes. We performed a similar analysis on all non-synonymous mutation features (i.e. regardless of whether the mutation is or is not in a cluster). Finally, we linked the multiscale mutation clusters to differential drug response using cancer cell line data. Fig 1 illustrates our approach on *PIK3CA* in breast invasive carcinoma, a prototypical example of how our method identifies multiple mutation clusters with differential associations with gene expression data. Additional details can be found in the Methods section and Supplemental Information.

### Characterizing multiscale clusters

M^2^C identified a total of 1255 multiscale clusters in 393 of the 549 genes analyzed. These genes were selected by taking the highest ranked genes in MutSig for each cancer type. The 156 genes without any clusters had all their mutations classified as uniform background noise by M^2^C and were omitted from further analysis. The following results indicate that our method finds multiscale regions of proteins which are enriched for mutations and frequently overlap with annotated protein domains. The multiscale clusters span a wide range of lengths: from 1 to 600 amino acids. Additionally, clusters have a highly variable number of mutations: 15 to 338 mutations. Finally, we note that each cluster is given a score which is the log of the ratio of its emission probability from its component of the mixture model to the emission probability under the null hypothesis that mutations are distributed uniformly across the gene. Higher scores indicate increased robustness as shown by cross-validation analysis (see Methods and S4 Fig). T2 in S1 Tables details pan-cancer cluster definitions, cluster scores, and overlapping protein domains.

We assigned clusters to specific tumor samples if there was at least one non-synonymous mutation in a sample at an amino acid position within a cluster. By combining tumor samples grouped by tumor tissue of origin, we were able to compare how clusters are assigned to different tumor types. T3 in S1 Tables details cluster assignments to tumor types. When clusters are assigned to specific tumor types, a high variability is seen in the way clusters are distributed between tumor types. On the high end, lung squamous cell carcinoma and uterine carcinosarcoma have over 80% of their non-synonymous mutations in clusters. On the low end, acute myeloid leukemia and thyroid carcinoma have 23% and 34% of their non-synonymous mutations in clusters, respectively. Neither the total number of non-synonymous mutations nor the total number of synonymous mutations is a good indicator for what percent of mutations are located within clusters in a specific tumor type. Interestingly, the ratio between the percentage of non-synonymous mutations in clusters and the percentage of protein sequence covered by clusters (which contain non-synonymous mutations) is much less variable; for the pan-cancer data set this ratio is 1.88 and when calculated between cancer types ranges from 0.88 (acute myeloid leukemia) to 2.23 (thyroid carcinoma). T4 in S1 Tables gives statistics on the distribution of clusters between different tumor types. Finally, we searched for tumor specific clusters by using Fisher’s exact test to determine if specific tumor types are enriched for specific clusters. We found 426 mutational clusters enriched for a specific tumor type at a false discovery rate of 1% and 996 at a false discovery rate of 10%. Cluster tumor type enrichment results are detailed in T5 in S1 Tables.

**Fig 2:**
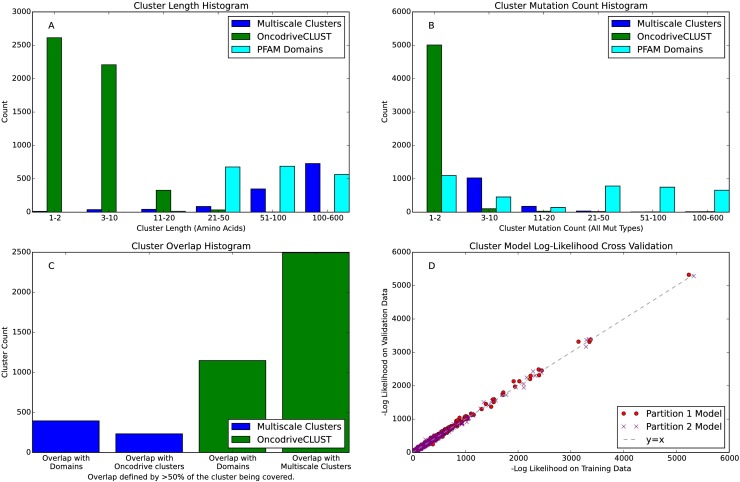
Cluster Statistics Comparing Multiscale Clusters from M^2^C with the DBSCAN based method OncodriveCLUST and Pfam domains. **A)** Cluster length histogram. **B)** Cluster mutation count histogram using all mutation types. **C)** Coverage with competing method histogram and Pfam. Cluster X is said to overlap cluster Y if over 50% of cluster X is covered by cluster Y. **D)** Cross-validation of mixture models: each circle shows the log-likelihood of the mixture model trained from partition 1 to generate the data from partition 2 for a single gene (red circles). The opposite analysis, using partition 2’s mixture model to generate data from partition 1, is also shown (purple x’s).

We compared the multiscale clusters to a Density Based Spatial Clustering of Applications with Noise (DBSCAN) based method called OncodriveCLUST and to protein domains from Pfam [24]. OncodriveCLUST’s approach also uses a kernel smoother to create a mutation density, albeit using only one predefined scale. Despite finding fewer clusters (OncodriveCLUST found 5185 clusters in 514 genes), we find that multiscale clusters tend to be larger and have more mutations. Additionally, multiscale mutation clusters cover (defined as >50% overlap) 48% of the alternative method’s clusters. On the other hand, OncodriveCLUST clusters cover only 18% of the multiscale clusters. Finally, we note that M^2^C has 9% more coverage by protein domains from Pfam compared to OncodriveCLUST (M^2^C has a total of 31% of multiscale clusters located within or overlapping with protein domains). These statistics are summarized in panels A-C of Fig 2.

We validated the M^2^C robustness by splitting our data set into two equally sized partitions and running the algorithm separately on each partition. We then compared how clusters from the different partitions overlap. We also make use the generative capabilities of the mixture models underlying our algorithm to use each partition’s model to predict the data from the other partition. In brief, we found that M^2^C robustness is dependent on mutation density; clusters with many mutations, regardless of size, tend to be validated across the two different partitions. Less populated small dense clusters are also well conserved. However, larger sparse clusters are more poorly replicated between partitions. Despite these differences, the mixture models generated by each partition do a very good job predicting the data set in the other partition (Fig 2D). For more details on our cross-validation tests, see the Methods section.

### Associating clusters with gene expression data

**Fig 3:**
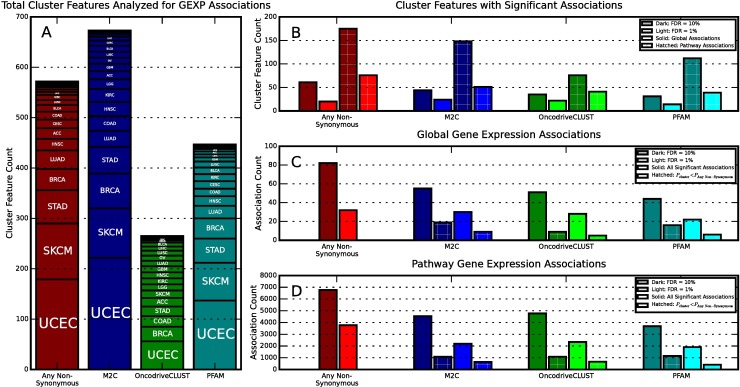
Gene Expression Association Statistics. **A)** The number of cluster features (meaning clusters in the context of a tumor type) analyzed for gene expression association broken down by tumor type and by clustering method. A single cluster can be analyzed multiple times if it is present with greater than 4 mutations in multiple tumor types. **B)** The number of cluster features with significant global gene expression associations (solid) and pathway gene expression associations (hatched). Two significance levels (1% is lighter colors and 10% darker colors) are shown for each method. Here a cluster feature with multiple associations is only counted once. **C)** The number of significant global gene expression associations found by each method at different significance levels (1% is lighter colors and 10% darker colors). Hatched bars indicate associations with lower P-values than the corresponding any non-synonymous feature for the same gene. Here cluster features with multiple associations are counted multiple times. **D)** Same as (C) but for pathway gene expression associations.

In order to begin to gain an understanding of the functional consequences of mutations in different clusters, we statistically associated gene expression data from TCGA by combining statistics from all gene expression data (i.e., global associations) and from subsets of gene expression data representing molecular pathways (i.e., pathway associations). The statistical associations were carried out on binary vectors that indicate whether a sample in a specific tumor type had a mutation in a specific cluster (1) or not (0). We refer to these binary vectors as cluster features. This association pipeline works by combining P-values from Kruskal-Wallis tests between gene expression data and cluster features with the Empirical Brown’s Method [25]. This methodology was chosen because, in general, gene expression from a particular pathway is not universally upregulated or downregulated due to cancer mutations. A summary of the results from these associations at different false discovery rates (FDR) can be seen in Fig 3. Further information on the association analysis can be found in the methods section.

In order to assess the robustness of our association methodology, we performed a cross-validation analysis. We measured two different robustness scores. “Association robustness” compares the associations between two data partitions using the same underlying set of clusters. “M^2^C plus association robustness” compares two data partitions each used to generate their own set of clusters which are subsequently analyzed for gene expression associations separately. Our analysis finds association robustness at 80% and M^2^C plus association robustness at 60%. This decrease is consistent with the observation that M^2^C robustness (described previously) is lower for lower scoring clusters, causing a decrease between partitions. However, higher scoring clusters tend to have stronger associations, making M^2^C plus association robustness higher than might be naively expected simply by combining M^2^C association robustness and association robustness. Further information on the cross-validation can be found in the Methods section.

We found that computing associations with gene expression comprises a complementary approach towards ranking cancer genes when compared with other methods such as MutSig [1]. We compiled a list of the top 67 genes from the MutSig rankings from across all cancer types that represent many of the most important cancer genes (see section B in S1 Text). At a FDR of 10%, 36% (a total of 24 genes) of the top genes contained at least one mutation cluster that is associated with global or pathway changes in gene expression in a specific tumor type.

Our results from the gene expression analysis highlight that clusters of many different length scales are associated with changes in gene expression, see S1 Fig. This corroborates the fact that functional regions of genes can range in size from single amino acids to multiple protein domains. As discussed later, these associations are often more specific than associations with any-non-synonymous mutation in the same gene. These results indicate the importance of being able to dynamically determine multiscale regions of interest within genes in order to better understand the spectrum of mutations underlying cancer.

Furthermore, we have found a number of cases where the association P-value between a cluster feature and gene expression data (pathway or global) is lower than the association P-value between the feature (variable) that encodes the occurrence of any non-synonymous mutation. These cases are of special interest because cluster features (i.e. variables that encode the presence or absence of a mutation in a particular cluster) are by definition subsets of the feature that encodes all non-synonymous mutations. This means that cluster features have fewer samples and thus less statistical power to detect associations with expression data. One would statistically expect them to have correspondingly higher P-values. However, in many instances we find that the opposite is the case. These decreased P-values provide additional evidence that mutation clusters can have specific functional consequences and provide a more nuanced view than considering an entire gene. A number of specific examples are highlighted below and a full list of these cases can be found in T6A and T7A in S1 Tables. The pathway association in T7A in S1 Tables also lists individual genes within a pathway with significantly differential expression between samples with a cluster feature and without. We have also computed pathway and global associations for samples which contain mutations not in any of our clusters. At a false discovery rate of 10%, about 90% of clusters are more strongly associated with a given pathway or global gene expression feature than mutations lying outside of all clusters. In some cases, this is an indication that our clusters are strongly associated with changes in gene expression. In other cases, the change in P-value can be attributed to the smaller sample size of mutations lying outside of all clusters. Therefore the comparison to the any non-synonymous feature, which will always have a larger sample size than the corresponding cluster feature, is more interpretable. A table of associations corresponding to the significant associations in T6A and T7A in S1 Tables for samples which do not contain mutations in any cluster can be found in T6D and T7D in S1 Tables. Note that associations were not computed if fewer than 5 samples in a given gene and tumor type had non-synonymous mutations outside of all clusters.

Finally, we uncovered a few instances where different cluster features in the same gene are associated at substantially different levels with gene expression data at the pathway or global level. These may be instances of multifunctional genes where mutations in different regions of the same gene have different functional consequences. In the following sections, we highlight specific examples found in our analysis. We note that the following discussion is not exhaustive and many more examples can be found by examining T6A S1 Tables (features associated with global changes in gene expression) and T7A in S1 Tables (features associated with pathway changes in gene expression).

As a further comparison to our method, we also analyzed global and pathway gene expression associations for OncodriveCLUST clusters and PFAM domains. Significant results for OncodriveCLUST can be found in T6B in S1 Tables (global) and T7B in S1 Tables (pathway). Significant results for PFAM domains can be found in and T6C in S1 Tables (global) and T7C in S1 Tables (pathway). Fig 3 compares the number of cluster features with significant gene expression associations and the total number of associations found by each method. At a false discovery rate of 1%, M^2^C finds a slightly higher percent of clusters with lower P-values than the corresponding any non-synonymous association when compared to OncodriveCLUST and PFAM domains, at 29%, 28%, and 22%, respectively. As the false discovery rate is increased, M^2^C continues to find a higher proportion of more strongly association clusters than OncodriveCLUST, despite typically finding fewer significant associations in total. Interestingly, at higher false discovery rates PFAM domains are the most likely to have a lower association P-values than the corresponding any non-synonymous association. The strong associations from PFAM domains are likely due to the overall large length and amino acid counts inside these domains which has the drawback that they contain much less positional information compared to the clustering methods. T8 in S1 Tables contains a more detailed set of statistics similar to those shown in Fig 3.

### Clusters associated with global changes in gene expression

**Fig 4:**
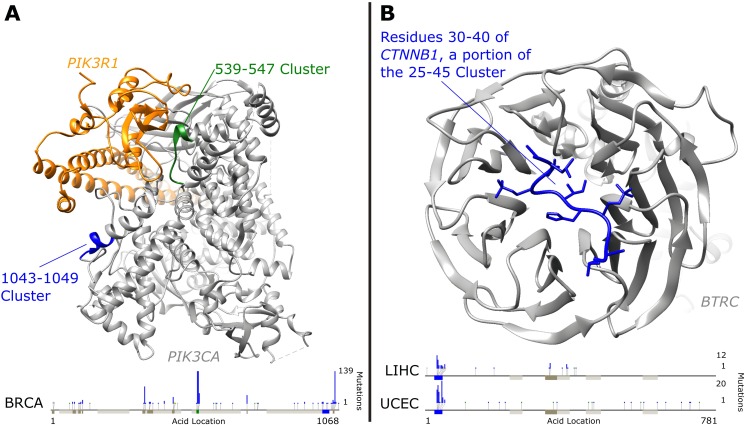
Clusters Highlighted in Protein Structures. **A)** PIK3CA (gray) bound to PIK3R1 (orange). PIK3CA has two clusters (539–547 in green and 1043–1049 in blue) with very different global gene expression association significance levels in Breast Cancer (BRCA) discussed in the text. **B)** Residues 30–40 of CTNNB1 (blue) bound to BTRC (gray). This region of Beta-catenin is inside the 25–45 cluster which contains degradation regulating phosphorylated amino acids and is strongly associated with global gene expression changes in uterine corpus endometrial carcinoma (UCEC) and liver hepatocellular carcinoma (LIHC). Bottom bars in both plots show linear protein sequences with additional clusters in dark gray and PFAM protein domains in light gray. Mutation count histograms are shown for specific tumor types above the sequence with green dots representing synonymous mutations, blue dots representing missense mutations, and yellow dots nonsense mutations. Protein images created using UCSF Chimera [26].

We identified highly cancer-related clusters in specific tumor types that are significantly associated with global changes in gene expression. In the cases of *PIK3CA* and *GATA3*, different clusters have widely different association levels with global changes in gene expression. This may be a statistical indicator of different mutation clusters within a single gene affecting the cellular environment in different ways. A full list of global gene expression associations can be found in T6A in S1 Tables. Here, we highlight several well-known and novel clusters in the cancer literature recovered by M^2^C:

Two of *PIK3CA’s* eleven mutation clusters are associated with global changes of gene expression in breast invasive carcinoma: amino acid positions 539–547 and 1043–1049. We note that the first clusters are significantly enriched for mutations in the following tumor types: breast invasive carcinoma, head and neck squamous cell carcinoma, uterine corpus endometrial carcinoma, cervical squamous cell carcinoma and endocervical adenocarcinoma (FDR < 1%). The second cluster is significantly enriched for mutations in breast invasive carcinoma and uterine corpus endometrial carcinoma (FDR < 1%). The 539–547 cluster contains three previously studied residues in an α-helical region which have been shown to increase the enzyme’s activity [22]. A hotspot point mutation at 1047 inside the 1043–1049 mutation cluster has been speculated to affect the position and mobility of the activation loop [27]. Interestingly, despite approximately 20% fewer mutations, the 539–547 cluster has a much lower global gene expression association P-value (about 6 orders of magnitude smaller), signifying that perhaps mutations in this cluster have different functional consequences. As seen in Fig 4A the 539–547 region of PIK3CA may be directly involved in binding to PIK3R1, providing another interpretation for the large association with changes in gene expression [28]. As one of the best studied cancer genes, it is unsurprising that many additional associations and cluster-tumor enrichments exist for PIK3CA which are detailed in our S1 Tables.

*ROBO3* has a cluster from 195–509 which overlaps with 4 lg-like C2 domains [29]. This cluster is associated with global changes in gene expression in uterine corpus endometrial carcinoma. The cluster is also enriched for mutations in lung adenocarcinoma (FDR < 5%), as well as in uterine corpus endometrial carcinoma (FDR < 1%). Previously, increased levels of *ROBO3* expression have been associated with metastasis in pancreatic cancer [30]. However, to our knowledge somatic mutations in this region have not been extensively studied. Furthermore, we note that *ROBO3* is ranked only 15540 in MutSig for uterine cancer [1]. Thus this gene might be a candidate for further study of its role in uterine corpus endometrial carcinoma and possibly the other tumor types mentioned above.

*GATA3* has a subset of three clusters in breast invasive carcinoma all containing predominantly nonsense (i.e. frameshift, indel, or stop) mutations: amino acids 325–334 with 15 mutations, amino acids 390–421 with 22 mutations and amino acids 429–443 with 15 mutations. We note that the most densely populated of these clusters occurs after the zinc finger binding domain while the first cluster occurs within the binding domain [29]. Interestingly, the 390–421 cluster is substantially more associated with global changes in gene expression than either of the other two clusters with a P-value nearly 2 orders of magnitude lower. Different *GATA3* mutations have been associated with Luminal A and B subtypes of breast cancer [31] and changes in survival prognoses [32]. We note that all three of these clusters are enriched for mutations in breast invasive carcinoma (FDR<1%).

### Clusters associated with pathway level changes in gene expression

We identified significant pathway associations with clusters in specific tumor types. These pathway associations go beyond simply implicating certain mutation clusters in cancer by shedding light on possible phenotypic effects of each cluster. At a false discovery rate of 10%, all of the genes associated with global changes in gene expression were also associated with at least one pathway (and usually with many pathways).This included 24 of 67 top ranked cancer genes from MutSig. These results are not surprising because each set of genes in a pathway is a subset of the global gene expression data. However, we note that by restricting our analysis to individual pathways, novel clusters associated with gene expression changes were detected indicating that this analysis is more nuanced. For a complete list of cluster pathway associations see T7A in S1 Tables. This table also included specific gene expression features in each pathway which are strongly associated with a mutation cluster. Below we discuss several examples of specific clusters which are associated with pathways but are not associated with global changes of gene expression.

*ZBTB20* has a cluster from 681–714. This cluster is associated with 9 pathways related to angiogenesis and regulation of the cell cycle in gastric adenocarcinoma. Previously single nucleotide mutations in *ZBTB20* have been associated with gastric cancer [33]. We hypothesize that a larger region of the *ZBTB20* gene as represented by this mutation cluster may be involved in gastric oncogenesis. Specifically, the frameshift deletions between the two zinc finger domains likely disrupt DNA binding and the C-terminal function of the protein. We note that this cluster is enriched in gastric cancer along with two other clusters in *ZBTB20*, 190–248 and 345–504 (FDR < 1%). The 190–248 cluster is also enriched for mutations in low grade glioma (FDR < 10%).

*PPP2R1A* has a cluster from 167–183 that overlaps a HEAT domain motif [34]. This cluster is associated with pathways related to cell differentiation and *MAPK* signaling in uterine corpus endometrial carcinoma. This gene has also been implicated previously in uterine and ovarian cancer [35]. This cluster is enriched for mutations in uterine corpus endometrial carcinoma and uterine carcinosarcoma (FDR < 1%). Additionally, the 237–275 in *PPP2R1A* is enriched for mutations in lung squamous cell carcinoma and gastric cancer (FDR<10%). The 391–490 cluster is enriched for mutations in uterine corpus endometrial carcinoma (FDR <1%).

*CHD4* is a chromatin helicase remodeling protein. It has a cluster from 945–1016. This cluster is associated with two pathways in uterine corpus endometrial carcinoma. One of these pathways is signaling events mediated by HDCA Class II, which is thought to form a complex which includes *CHD4* [36]. The above selection of examples is by no means exhaustive. Additional examples can be found and investigated in more depth by examining T7A in S1 Tables.

### Clusters can produce stronger pathway and global gene expression associations

We identified statistically significant clusters in specific tumor types with a lower combined P-value across all gene expression features than the corresponding any non-synonymous mutation feature in a specific tumor type. These results are annotated in T6A in S1 Tables. As examples, this analysis picks up two well-known mutation sites. Our algorithm detected a cluster in *BRAF* from amino acids 600 to 601 which is more significantly associated with global changes in gene expression (P<10^−13^ with 106 non-synonymous mutations) in skin cutaneous melanoma despite having fewer mutations than the any non-synonymous feature (P<10^−10^ with 126 total non-synonymous mutations across the entire gene). Similarly, in thyroid carcinoma the 600–601 mutation cluster has 235 non-synonymous mutations and is more significantly associated to global changes in gene expression (P<10^−82^) than any-non-synonymous mutation which has 237 total non-synonymous mutations (P<10^−80^). These are common mutations previously implicated in cancer [37].

Additionally, the 25–45 amino acid region of *Beta-catenin* (*CTNNB1)* is found to be more significantly associated with global changes in gene expression (P<10^−24^ with 67 non-synonymous mutations in the cluster) than all non-synonymous mutations in the gene (P<10^−21^ with 80 total non-synonymous mutations in the gene) in uterine corpus endometrial carcinoma. A similar result is seen in liver hepatocellular carcinoma where the cluster is more significantly associated with global change in gene expression (P<10^−14^ with 37 non-synonymous mutations in the cluster) than all non-synonymous mutations in the gene (P<10^−12^ with 53 non-synonymous mutations). This cluster is also enriched for mutations Adrenocortical carcinoma (FDR < 5%), uterine corpus endometrial carcinoma (FDR < 1%) and in liver hepatocellular carcinoma (FDR < 1%). *Beta-catenin* is known to be implicated in numerous types of cancer [38]. This region corresponds closely to a region of phosphorylated peptides along the *CTNNB1* chain which are known to regulate the degradation of *CTNNB1* [39,40]. Fig 4B shows the structure of the N-terminus region of *Beta-catenin* located within the 25–45 amino acid cluster bound to the WD40 domain of *β-TrCP1 (BTRC)* [41]. Mutations in this cluster are likely to affect this binding and thereby the regulation of *Beta-catenin*. The fact that this region is not an annotated protein domain illustrates the flexible nature of M^2^C in picking out different kinds of functional regions of interest.

We also identified cluster pathway associations in specific tumor types with lower pathway-cluster P-values than the corresponding any non-synonymous mutation feature in the same tumor type. These results are annotated in T7A in S1 Tables. One example is the 248–254 cluster in *FGFR3* in bladder urothelial carcinoma. Point mutations in this region have been previously implicated in low grade glioma tumors [42]. We note that mutations in this region are more significantly associated with 10 pathways than the any non-synonymous feature. These pathways are involved in a large number of molecular processes ranging from cell cycle control to *Reelin* signaling. This suggests diversity in the role of *FGFR3* as an oncogene in bladder cancer. This cluster is also enriched for mutations in both bladder cancer (FDR < 1%) and lung squamous cell carcinoma (FDR < 10%). Another example is the 88–98 cluster in *PGM5* in gastric adenocarcinoma which is more significantly associated with 12 pathways when compared to any non-synonymous mutation in the same gene. We note that this cluster is enriched for mutations in gastric cancer (FDR < 1%) and the 456–522 cluster is enriched in lung adenocarcinoma (FDR < 5%). Although *PGM5* has been ranked as a possible cancer gene according to MutSig, to the best of our knowledge this particular region has not been studied.

These cases demonstrate how decreasing sample size by considering a specific cluster as opposed to a specific gene can provide a more nuanced lens for finding statistical associations and aid in inferring the functional consequences of mutations. This occurs because clusters can represent functional regions of a gene and thereby limit the analysis to mutations within that region. This spatial specificity has the effect of excluding background mutations outside the cluster from the analysis.

### Pathway associations reveal differential functions of mutation clusters in the same gene

**Fig 5:**
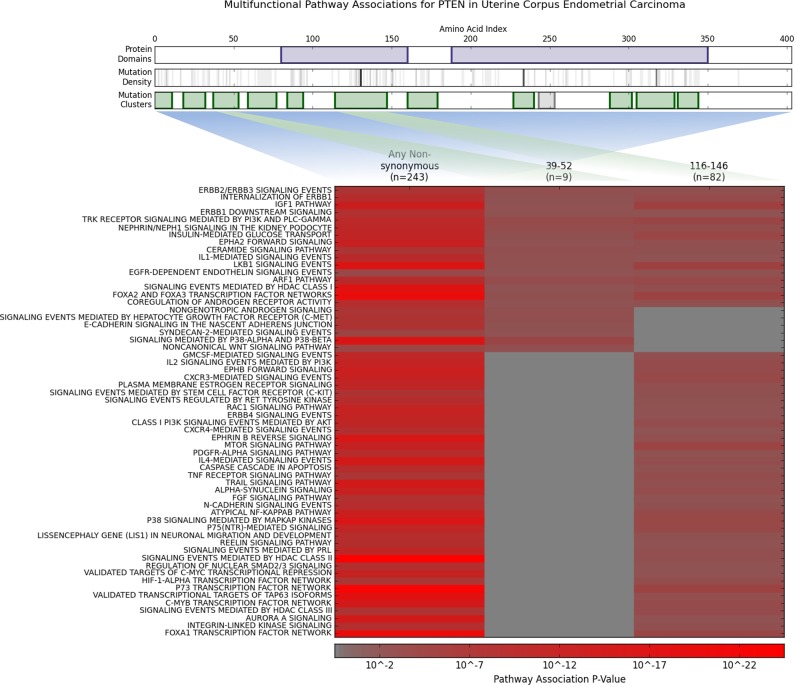
Differential Pathway Associations for PTEN clusters in Uterine Corpus Endometrial Carcinoma. Clusters without significant pathway associations are omitted for clarity. A false discovery rate of 1% was used to filter for significance.

*PTEN* has two clusters that are associated with global changes in gene expression in uterine corpus endometrial carcinoma. The first of these clusters from 39–52 has 9 mutations, 8 of which are nonsense mutations. The second cluster from 116–146 has 74 mutations which has 66 missense and 16 nonsense mutations. This cluster encompasses the P-loop of the protein [43]. Additionally the first cluster is associated with 16 pathways including 5 not associated with the second cluster. Similarly, the second cluster is associated with 51 pathways including 40 not associated with the first cluster. The differential associations between cluster features found in *PTEN* are visualized in Fig 5.

*FUBP1* has two clusters each with predominantly nonsense mutations with differential pathway association in lower grade glioma. The 88–206 cluster overlaps well with a KH-1 domain which is involved in RNA and DNA binding [44]. The second cluster does not overlap with any known domains [29]. One possible explanation is that nonsense mutations earlier in the protein sequence result in total loss of function while nonsense mutations later in the sequence only effect the end of the protein structure. This could result in differential effects from mutations in these two clusters. See S2 Fig for an association heatmap.

*E-Cadherin* has 3 clusters with differential pathway associations in breast invasive carcinoma. All these clusters have predominantly nonsense mutations. Of particular interest are the second two clusters. The cluster from 144–222 occurs at the beginning of the first cadherin domain in the protein and the cluster from 644–733 occurs towards the end of the last (fifth) cadherin domain in the protein [45]. It is possible that the 144–222 cluster interferes with the ability of the protein to insert itself into the cellular membrane. On the other hand, the 644–733 cluster may not interfere with membrane insertion and only affect the cytoplasmic part of the domain. See S3 Fig for an association heatmap.

### Mutation clusters help to explain differential drug response in cancer cell lines

After having established that the location of a mutation in a gene, i.e. in a cluster identified by M^2^C, is associated with differences in gene expression, we aimed to establish whether mutation clusters could be therapeutically relevant. To this end, we applied the mutation clusters to a large cancer cell line drug screening effort; the Genomics of Drug Sensitivity in Cancer (GDSC) [46,47]. We chose to use drug response measurements in cancer cell lines as an indication of the functional importance of the mutation clusters.

Data obtained from GDSC included the drug response to 142 anti-cancer drugs across 714 cell lines as well as non-synonymous mutation calls for 77 known cancer genes across these cell lines. First, we established that the clusters identified using M^2^C on the TCGA data were enriched for mutations found in the cell lines. Specifically, for the 17 genes with at least 10 mutations across the cell lines, 14 showed strong enrichment of mutations in the M^2^C clusters (p-value ≤ 0.05, FDR ≤ 5%, T12 in S1 Tables). For 12 of these genes, there were at least 5 cell lines that had a mutation in one of the mutation clusters that we identified using M^2^C. We performed association tests between the drug response for each of the drugs, as expressed in IC50 values, and the mutation clusters with at least 5 mutations as well as the any non-synonymous mutation feature for these 12 genes. See Methods section for details.

Using an FDR of 10% we found 176 significant associations between the drug response of a drug and either a mutation cluster (64) or the any non-synonymous mutation feature (112) of a gene. Significant associations were found for all 12 genes across 77 of the 142 drugs. There were 130 gene-drug combinations, where either or both a mutation cluster and the any non-synonymous mutation feature of the gene were associated with the response to a drug. Interestingly, of these 130 cases, there were 35 (27%) gene-drug combinations where a mutation cluster in the gene showed a stronger association with drug response than the any non-synonymous mutation feature (T13 in S1 Tables).

**Fig 6:**
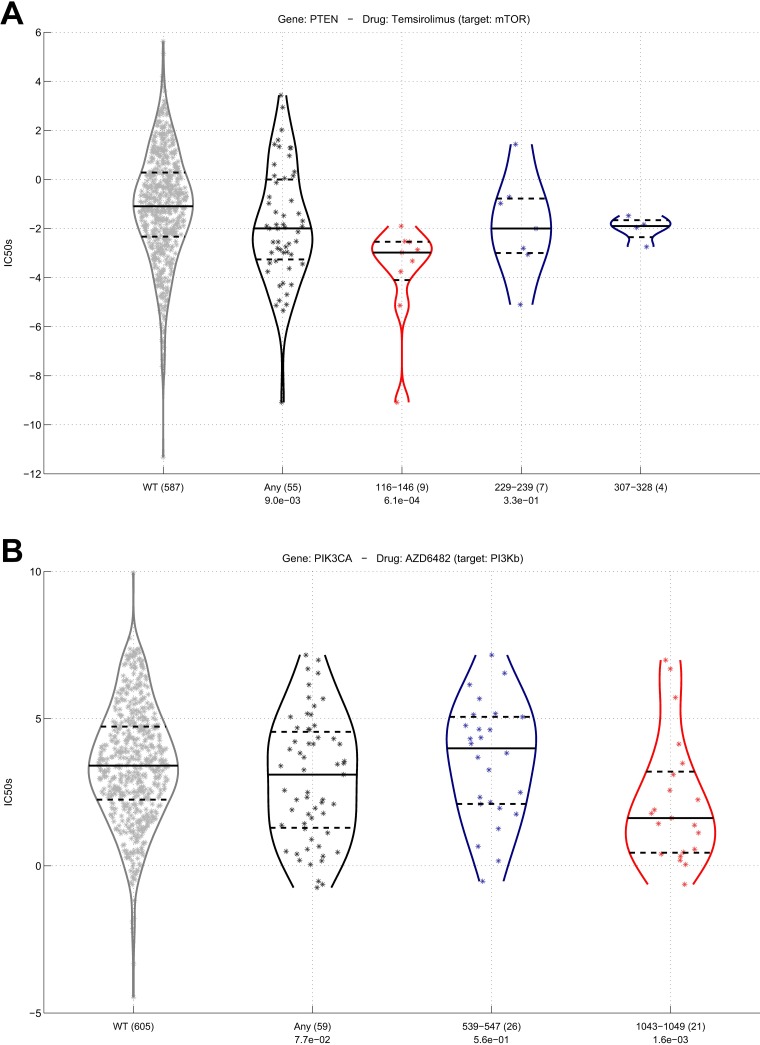
**A)** Violin plot of the response of mTOR inhibitor Temsirolimus (IC50s, y-axis) across cancer cell lines. The cell lines are grouped (x-axis) as wild-type PTEN (WT), depicted with gray markers and gray violin outline, cell lines with any non-synonymous mutation in PTEN (Any), depicted in black, and cell lines with a mutation in one of the hotspots, depicted in blue. Mutation clusters and any non-synonymous mutation features that are significantly associated with drug response (FDR<10%) are depicted in red. **B)** Violin plot of the response of a PI3Kb inhibitor for mutation clusters in PIK3CA.

Amongst these 35 cases, we found many known activating hotspot mutations of genes in the *MAPK* pathway. Specifically, there are 10 drug associations with the *BRAF* mutation cluster 600–601, 6 with *NRAS* mutation cluster at amino acid 61 and 4 with *KRAS* mutation cluster 12–13. These well-known examples can be interpreted as a positive control of the importance of considering mutation clusters compared to the mutations across the whole gene. Additionally, there are 5 drug associations with the various mutation clusters that we found for tumor suppressor *TP53*. This is consistent with *TP53* being the most prominent tumor suppressor gene. In Fig 6 we highlight two examples where drug response is more strongly predicted by a mutation cluster than by any non-synonymous mutation in the same gene. First, we observed that cell lines with mutations in the cluster 116–146 in *PTEN* strongly responded to mTOR inhibitor Temsirolimus, whereas mutations across the entire gene showed only a small indication of drug sensitivity (Fig 6A). Second, we observed that cell lines with mutations in the cluster 1043–1049 in *PIK3CA* showed a stronger response to a *PI3K* beta inhibitor than other mutation clusters or the any non-synonymous mutation feature (Fig 6B). This corresponds with our observation that in breast cancer this same mutation cluster is very strongly associated with global changes in gene expression compared to other clusters in *PIK3CA*.

These results provide additional support for the idea that it is important to consider mutations in a *specific* region of a cancer gene, and not merely a single amino acid alteration or a mutation anywhere in the entire gene.

## Conclusion

We have identified multiscale mutation clusters in genes using a pan-cancer data set. Previous approaches have focused on short (frequently single) amino-acid hotspots [7] as well as identifying globally mutation enriched cancer genes [1]. Our method (M^2^C) dynamically combines these two approaches by searching for variable-length regions of interest within individual genes. We have shown that M^2^C clusters are indeed multiscale covering a wide range of length scales and can be detected in genes with variable mutation density. These clusters frequently overlap with protein domains, but also cover regions where no known domain exists. We show that many of the clusters found by M^2^C are representative of functional regions of proteins where mutations have a larger effect in terms of influencing the hallmarks of cancer [48]. M^2^C represents a data driven approach towards systematically identifying regions of interest inside of genes with many areas for further investigation and improvement. Firstly, any statistical algorithm will only improve in accuracy as newer and larger data sets are generated. As more cancer tumors are sequenced, we expect our method to detect more mutation clusters, in particular novel clusters in under-studied cancer genes. Secondly, our mutation clustering methodology is sequence based; we suspect that a three dimensional version of such a multiscale algorithm–which implicitly takes protein structure into account–would result in an improvement in identifying functional mutation clusters. Approaches to detect mutation hotspots in 3D structures already exist [49] but they are not based on a multiscale framework. It is likely that many of our one-dimensional clusters, if mapped onto the 3D structure of a protein, would merge together. A future direction is to carry out this mapping and determine more realistic structural mutation clusters. We also suspect that such an approach would further increase statistical power.

In order to identify important clusters and shed light on their function, we examined cluster gene-expression associations both globally and on a pathway level. In this analysis, we have highlighted a number of clusters that we believe are strongly implicated as driver clusters in diverse tumor types due to global gene expression associations. Additionally our pathway level of analysis has helped reveal more specific functional associations between mutation clusters and gene expression. Furthermore, we have shown that certain genes contain multiple clusters that may have different functional consequences, suggesting that different mutations in these genes may play different roles in cancer onset and progression based upon the location of the mutation. Finally, we investigated whether mutation clusters are associated with drug response data in cancer cell lines and found many mutation clusters which are associated with differential drug sensitivity. These findings emphasizes the importance of taking a flexible approach in terms of identifying genomic features–specifically that neither an entire gene approach nor a single amino acid approach is necessarily sufficient.

### Availability

In order to facilitate exploration of our data, including mutation clusters and pathway associations, we have created an interactive graphical website: m2c.systemsbiology.net. The multiscale mutation clustering algorithm has also been made publicly available: https://github.com/IlyaLab/M2C. All significant results from our pipeline (including those from other methods) can be found as a multi-tabbed excel document S1 Tables. These same tables are also available as TSV’s from our website. Detailed descriptions of the tables and the data they contain are in S1 Table Descriptions.

## Methods

### Identifying multiscale clusters

An initial list of 628 genes was compiled by taking the highest ranked genes from MutSig [1], i.e. the most significantly frequently mutated genes, using a q-value threshold of 0.1. (The MutSig results that we used in this work are based on the October 2014 analysis run of the Broad Institute TCGA Genome Data Analysis Center Firehose pipeline, available through http://ezid.cdlib.org/id/doi:10.7908/C1K64H78 and http://gdac.broadinstitute.org/runs/analyses__2014_10_17/data/). These genes were further filtered to ensure that there are in total at least 15 mutations in each gene across all 23 cancer types considered. This resulted in a list of 549 genes on which we ran our multiscale clustering algorithm. The cancer types considered are: ACC, BLCA, BRCA, CESC, COAD, GBM, HNSC, KICH, KIRC, KIRP, LAML, LGG, LIHC, LUAD, LUSC, OV, PRAD, READ, SKCM, STAD, THCA, UCEC, and UCS. Full names can be found in T1 in S1 Tables. The raw mutation data we used for clustering can be found in T10 in S1 Tables.

Clusters are identified using all mutations combined from the pan-cancer data set to increase statistical power. These files are created from TCGA mutation annotation format (MAF) files and annotated with Annovar (section B in S1 Text) [50].

**Fig 7:**
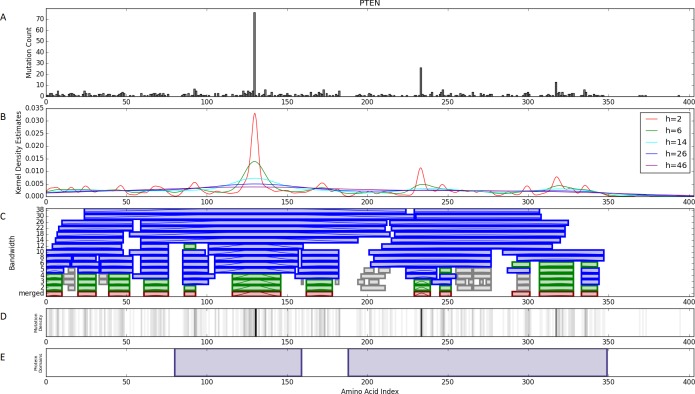
Multiscale Information-Based Clustering Algorithm. **A)** Pan-cancer mutation data is merged across all 23 tumor types for a single gene (PTEN). **B)** Gaussian kernel density estimates smooth this data at 28 different bandwidths or scales (a limited selection is shown for clarity). **C)** Each kernel density estimate is used to seed a multivariate mixture model of normal distributions and a single uniform distribution to represent background noise. Initial guesses for the locations of the normal distributions are determined from the local maxima of the kernel density estimates. Clusters from the mixture models (blue) are merged together using the greedy algorithm resulting in a final set of multiscale clusters (red). Green are duplicates of the red clusters and shown to clarify the process. Grey bars are excluded due to too few mutations. **D)** A mutation spectrum for PTEN. **E)** The two annotated protein domains in PTEN from PFAM.

Our multiscale mutation clustering algorithm (M^2^C) identifies mutation clusters at multiple scales. Each scale represents different sized genetic features. First, M^2^C converts TCGA mutation calls in amino acid space from all 23 cancers into multiple continuous probability density functions (Fig 7A and section C step 1 in S1 Text). This smoothing is done using a kernel density estimate (KDE) with a Gaussian kernel at 28 different bandwidths between 2 and 450 (amino acids units). Each bandwidth represents a different length scale of amino acid features ranging from single amino acids to entire protein domains (Fig 7B and section C step 2 in S1 Text). These KDEs are each used to seed a multivariate mixture model consisting of *n* Gaussians and 1 uniform distribution, where *n* is the number of local maxima in a given KDE. The noise weight is initially estimated by the fraction of synonymous mutations in the gene. The means of each Gaussian are initialized as the locations of the local maxima of the KDE. The standard deviation of each Gaussian is set as the distance between the two adjacent local minima around a given maxima. Finally, the weight of each Gaussian in the mixture model is estimated by the density at the local maxima minus one-n^th^ of the noise weight (section C step 3 in S1 Text). An expectation maximization (EM) algorithm then optimizes the mixture model (Fig 7C blue bars). This process results in a set of clusters for each scale.

In order to generate a single set of multiscale clusters, the mixture models from each different scale are merged using a greedy algorithm. The clusters resulting from the EM algorithm for each scale with at least 15 mutations are placed in binary tree using Scipy’s hierarchical clustering function (section C step 4 in S1 Text). The cutoff of 15 was chosen to ensure that clusters had sufficient mutations for further statistical analysis. The unsigned area between the two Gaussian curves is used as the distance metric. The binary tree is flattened using a recursive greedy algorithm to minimize the Akaike Information Criteria with finite size correction (AICc) [51]. In short, given any two sets of clusters the algorithm finds the set of non-overlapping clusters which minimize the AICc. Although the enumeration of all non-overlapping sets of clusters is a computationally costly problem, by placing the clusters in a tree and performing this enumeration recursively the scale of the problem is reduced (section C step 6 in S1 Text). M^2^C results in a single set of non-overlapping multi-scale clusters (Fig 7C red bars). We refer to clusters as a pair of amino acid positions *x-y* with *x* being the start of the cluster and *y* being the end of the cluster.

### Scoring M^2^C clusters

The score, *S*_*c*_, of cluster *c*, is given by the log of the ratio of the emission probability of the mutations in the cluster with the emission probability of the same mutations based upon the null hypothesis of a uniform mutation distribution across the cluster,

$$S_{c}=\sum_{i=1}^{N}\log\frac{G(M_{i};\;\mu_{c},\;\sigma_{c})}{U}.$$

*M* is the set of all *N* pan-cancer mutations in cluster c. *G(x; μ*_*c*_, *σ*_*c*_*)* is the normalized Gaussian distribution with mean μ_c_ and standard deviation σ_c_ representing the unweighted component of the mixture model corresponding to cluster *c*. Finally *U = L*^*-1*^ is the emission probability of single mutation by a uniform distribution over the gene containing the clusters of length *L* (in amino acids).

### Multiscale mutation algorithm cross-validation analysis

In order to test the robustness of the M^2^C algorithm (referred to as M^2^C plus robustness), we split the entire underlying mutation data into two partitions and used each partition to generate a new mixture model for each gene. We then compared the resulting clusters from each partition. First, we compared the log-likelihoods of the mixture model on the training data (i.e. data from the partition which generated the model) to the log-likelihood of the mixture model on the validation data (the other partition). The log-likelihoods of the two models are highly correlated; Spearman correlation = .99, P≈0. These results are plotted in Fig 2 and indicate that the statistical model underlying M^2^C is robust. We also calculated the percent of clusters conserved between the two partitions. We define ‘conserved’ to mean that for two clusters in the same gene from different partitions, one of the clusters overlaps the other by at least 50%. Our cross-validation analysis shows that on average M^2^C robustness is about 40%, meaning about 40% of clusters are conserved. However, we further note that smaller denser clusters are more highly conserved and overlap by a greater percentage between partitions than large sparse clusters. The cluster score provides a good indication of how robust a cluster is likely to be, as shown in S4 Fig.

### Characterizing clusters

We compared M^2^C to a different greedy clustering algorithm that had already been applied to TCGA data [7]. We ran the alternate method on the same data using default parameters and compared the resulting clusters to the multiscale clusters by looking at length, mutation counts, and how the different clusters overlap with each other and annotated protein domains from PFAM [34].

### Cluster cancer type enrichment analysis

To determine whether specific cancer types are enriched for specific mutation clusters, we used Fisher’s exact test to calculate an enrichment P-value. A contingency table for each cluster cancer type pair was created across the pan-cancer set of samples based upon the two Boolean variables: 1) Is the sample inside the cluster and 2) Does the sample belong to the cancer type being analyzed. These results were then tested for significance together using the Benjamini–Hochberg method [52] with results at false discovery rates of 1%, 5%, 10% and 25% reported in T5 in S1 Tables.

### Integrating clusters with gene expression data

We created 23 binary feature matrices, one for each cancer type. A binary feature matrix represents which tumor samples contain mutations in which clusters. A tumor sample is said to be positive (1) for a given cluster if it contains a non-synonymous or nonsense mutation within the cluster (see boxes B and C of Fig 7 for an illustration). Additionally we defined an ‘any non-synonymous mutation feature’ for each gene. A gene is said to have any non-synonymous mutation if it contains a non-synonymous mutation inside or outside of a cluster.

We used the binary feature matrices to compare cluster assignments within tumor samples to gene expression data from the TCGA. This analysis was carried out separately for each cancer type. We used a Kruskal-Wallis test between features in the binary feature matrix and 2194 gene expression features. These features were chosen based upon the genes in the NCI pathway interaction database (PID) [53]. In each test, we used data from all tumors in each cancer type. Cluster features and any non-synonymous features with fewer than 5 positive tumor samples were excluded from this analysis. This analysis resulted in a set of P-values representing associations between cluster features and gene expression levels.

### Gene expression pathway analysis

**Fig 8:**
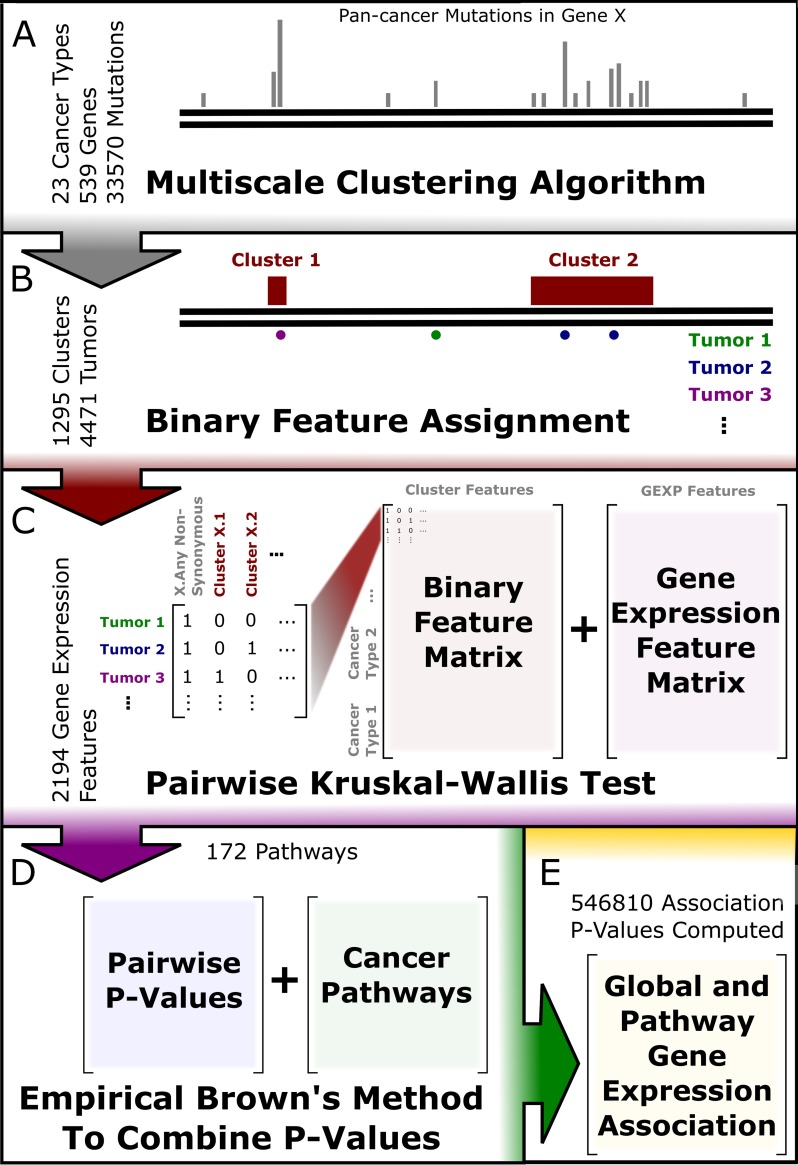
Statistical Methods Pipeline. **A)** 549 genes with a total of 33507 pan-cancer mutations are run through our multiscale clustering algorithm resulting in 1295 clusters. **B)** Clusters are assigned to 4471 tumors samples across 23 tumor types creating a binary feature matrix. A tumor sample is said to be positive for a cluster if there is any non-synonymous mutation in the tumor and the cluster. **C)** The binary feature matrices are statistically compared to 2194 gene expression features separately for each cancer type using the Kruskal-Wallis Test. **D)** The pairwise P-values from the Kruskal-Wallis tests are combined globally and on the pathway level using the Empirical Brown’s Method across 172 Pathways. **E)** This resulted in 546810 association P-values.

In order to improve statistical power and gain more detailed understanding of how clusters affect molecular pathways, we combined P-values from the gene expression cluster associations. To combine P-values we made use of the Empirical Brown’s Method for combining dependent P-values by taking into account the mutual correlation between genes in a pathway [25]. First we combined all 2194 gene expression associations for each cluster. We call these combined P-values global gene expression P-values. We then combined gene expression association P-values from each pathway in the PID. This resulted in a set of cluster pathway association P-values. We carried out significance testing on the global and pathway associations separately using the Benjamini–Hochberg method [52]. Fig 8 shows a schematic for the complete data processing pipeline.

### Gene expression pathway association cross-validation

In order to ensure that our gene expression association pipeline is robust, we performed a cross-validation analysis. We used this analysis to calculate to robustness metrics. Association robustness measures the robustness of the gene expression association pipeline independently of the clustering pipeline. M^2^C plus association robustness measures the gene expression association pipeline combined with the M^2^C algorithm. First, we selected a subset of our data corresponding to all gene-tumor type pairs with significant global or pathway associations. This subset is made up of 369 significant pairs from 226 genes across 22 tumor types. For each of these gene-tumor type pairs, we independently partitioned the entire data set into two partitions each corresponding to an equal number of samples across all 23 cancers. This partitioning was done ensuring that the number of non-synonymous and synonymous mutations was about equal between partitions. We then re-ran M^2^C on each partition, resulting in two sets of clusters. For each set of clusters, we projected data from the training partition and the validating partition (only considering one specific tumor type) to generate binary cluster features. We ran these features through our gene expression association pipeline and carried out significance testing at a false discovery rate of 10%. To calculate association robustness, we compared significant associations from the two partitions projected onto the same set of clusters and found that 85% of significant associations were conserved between partitions. In addition, we found a high correlation between the association log P-values between conserved significant associations between partitions: spearman correlation = 0.76, P<10^−73^. To calculate M^2^C plus association robustness, we further compared associations between different sets of clusters (each generated by a different partition). For this comparison, we projected the data from a given partition onto the corresponding clusters to generate feature vectors which were used for the gene expression association analysis. An association is said to be conserved if that association is significant (at FDR = 10%) for two clusters in the same gene where either cluster covers the other cluster by at least 50%. Between separate sets of clusters, we found that 63% of associations were conserved. The conserved associations also showed a high degree of correlation in their log P-values: spearman correlation = .87, P<10^−56^. Cross-validation data is included graphically in S5 Fig.

### Calculating significant single gene expression features

In the pathway association table (T7A in S1 Tables), we have included lists of significantly differentially expressed gene expression features within a pathway that are strongly associated to a cluster (or any non-synonymous mutation). To determine if a gene expression feature is upregulated or downregulated, we compared the mean gene expression for samples which contained a non-synonymous mutation in that cluster (or any non-synonymous mutation) to the mean gene expression for samples which do not contain such a mutation. We used the Kruskal Wallis test P-values we had already computed in our analysis pipeline to determine significance, correcting for multiple testing separately for every pathway using the Benjamini–Hochberg method at a false discovery rate of 1% [52].

### Drug response analysis of cancer cell lines

We used a slightly expanded version of a previously published cancer cell line dataset [46,47]. Specifically, the dataset contained 714 cell lines and 142 anti-cancer drugs. The half-maximal inhibitory concentrations at 72 hours (IC50s) obtained in this dataset were used to represent the drug response. In this work, IC50s were recorded as the natural logarithm of the half-maximal inhibitory μM concentration. The drug screening dataset is incomplete, i.e. not all 142 drugs have been screened across all 714 cell lines. In total 81,700 IC50s were measured and 19,688 (19%) were missing values. The statistical tests were applied to each drug separately; cell lines that lack an IC50 were not used. We did not impute missing IC50s. This cell line dataset contains non-synonymous mutation calls for 77 known cancer genes across these cell lines based on capillary sequencing. Additional information on these data is found in [46,47] and on the Genomics of Drugs Sensitivity in Cancer webpages (http://www.cancerrxgene.org/). The data as used in this manuscript can be found in T11 in S1 Tables.

For each of the genes, we created a binary ‘any non-synonymous mutation’ feature, which was 1 for all cell lines with a non-synonymous mutation in the gene and 0 otherwise. Also, for each of genes we created binary mutation cluster features using the clusters identified by M^2^C on the TCGA data. Specifically, the binary mutation cluster feature is 1 when the cell line has a non-synonymous mutation in the cluster and 0 otherwise. We discarded all features that had less than 5 1’s. Then, for each combination of a binary mutation feature and a drug we applied a simple Kruskal-Wallis test to test whether the group of cases (1’s in the binary mutation feature) had an equal median IC50 compared to the control group (0’s in the binary mutation feature). Low P-value indicates a significant difference in the median IC50s between the two groups. P-values for all combinations are given in T13 in S1 Tables. Results were considered significant at a FDR of 10%.

## Supporting information

S1 Fig Percentage of clusters with at least one significant gene expression association. Pathway and global associations are both shown binned by cluster size. Under the cluster size, n signifies the total number of clusters in each bin. Four different false discovery rates are shown (1%: Red, 5%: Cyan, 10%: Green, 25% Blue).
(TIF)

S2 Fig Differential Cluster Pathway Associations in *FUBP1* in Brain Lower Grade Glioma. Clusters without significant pathway associations are omitted for clarity. A FDR of 1% was used to filter for significance.
(TIF)

S3 Fig Differential Cluster Pathway Association in *CDH1* in breast invasive carcinoma. Clusters without significant pathway associations are omitted for clarity. A FDR of 1% was used to filter for significance.
(TIF)

S4 Fig Multiscale Mutation Clustering Cross-validation Heatmaps. **Left)** Average overlap percentage for clusters from two partitions (binned by length and mutation count). Gray boxes contain no items. Number of clusters in each bin is indicated by n. **Right)** Average cluster score for the same binned clusters showing that this score is a reasonable proxy for robustness.
(TIF)

S5 Fig Gene Expression Pathway Association Cross-validation Scatter Plots. **Left)** This plot shows association robustness. Data was separated into two partitions A and B. Data from A was used to generate the clusters (“training partition”). Data from B (the validation partition) is compared to A by projecting each partition separately onto the same set of clusters and comparing the pathway associations. This process was then repeated with using B as the training partition and A as the validation partition on a different set of clusters. **Right)** This plot shows M^2^C plus association robustness. Here, partition A and partition B were both used to generate separate sets of clusters and the downstream association analysis was performed independently. Cluster associations are matched if the one of the two clusters (from partition A and B respectively) overlap the other by at least 50%.
(TIF)

S1 Tables All Supplemental Tables. This document includes all the supplemental tables referenced in the manuscript as separate excel tabs. Detailed descriptions of these tables can be found in S1 Table Descriptions document. The tables are also downloadable as individual TSV’s from the M^2^C website, http://m2c.systemsbiology.net/.
(XLSX)

S1 Table Descriptions Descriptions of all supplemental tables. This document contains descriptions of the supplemental tables, including specific break downs of what information is in each table and how it is formatted. The actual data can be found in S1 Tables as a single excel spreadsheet or as individual TSV’s from http://m2c.systemsbiology.net/.
(DOCX)

S1 Text Data, Methods, and Algorithm Details. This document contains detailed information on where the data used in this work comes from, data processing steps, and an in-depth description of the M^2^C algorithm.
(DOCX)
