# Correction: Multiscale mutation clustering algorithm identifies pan-cancer mutational clusters associated with pathway-level changes in gene expression

**DOI:** 10.1371/journal.pcbi.1005472

**Published:** 2017-04-06

**Authors:** The PLOS Computational Biology Staff

The funding statement for this article should read as follows:

“This work was supported by grants U24CA143835 and P01-CA077852-16A1 from the National Cancer Institute, http://www.cancer.gov/, and grant P50GM076547 from The Center For Systems Biology, http://www.centerforsystemsbiology.org/. The funders had no role in study design, data collection and analysis, decision to publish, or preparation of the manuscript.”
