# Supplementary figures and images for: Multiscale mutation clustering algorithm identifies pan-cancer mutational clusters associated with pathway-level changes in gene expression
Source: PLoS Comput Biol. 2017 Feb 7;13(2):e1005347. doi: 10.1371/journal.pcbi.1005347 (PMC5321471; doi:10.1371/journal.pcbi.1005347)

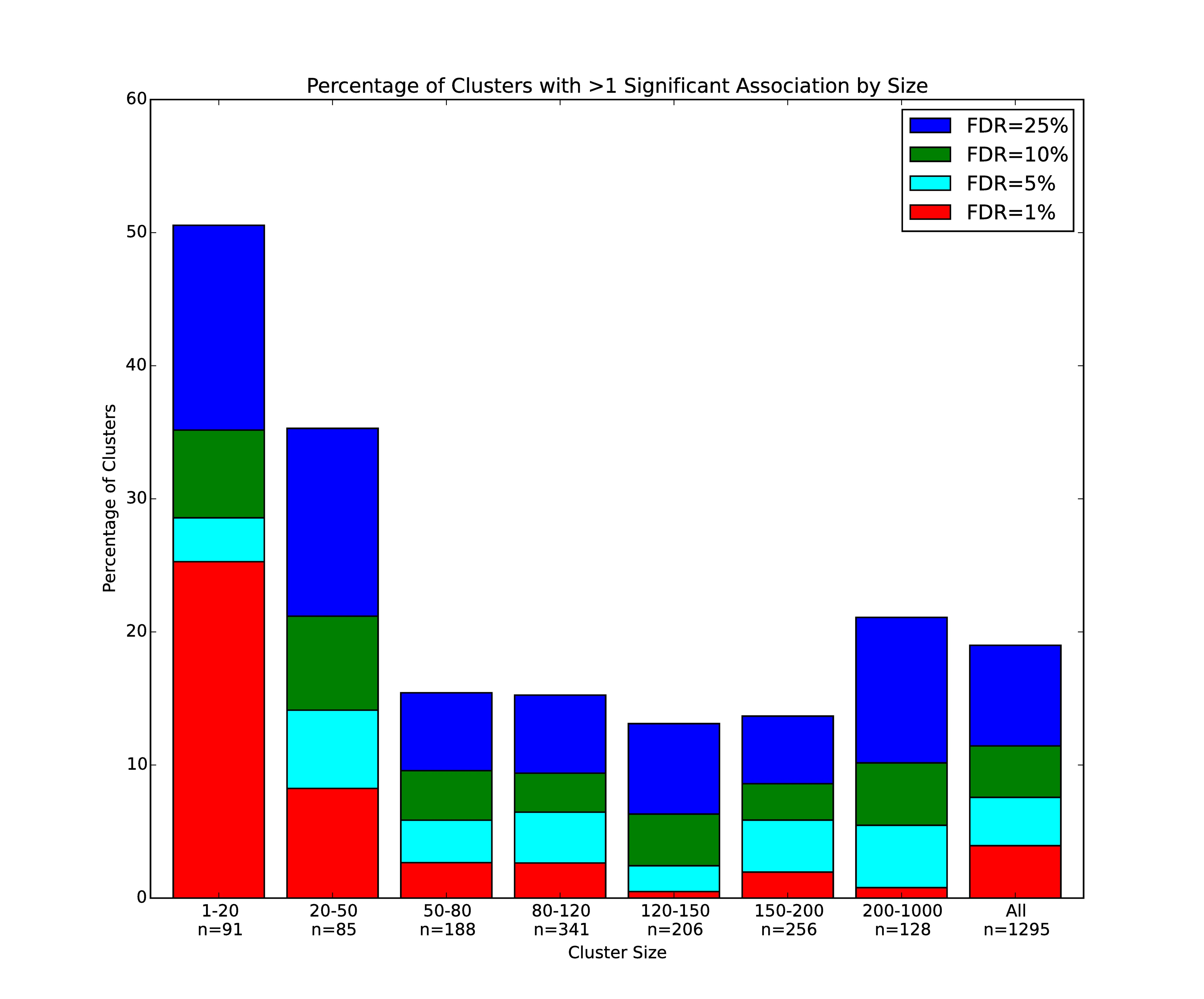

Supplement: S1 Fig — Pathway and global associations are both shown binned by cluster size. Under the cluster size, n signifies the total number of clusters in each bin. Four different false discovery rates are shown (1%: Red, 5%: Cyan, 10%: Green, 25% Blue). (TIF) [file pcbi.1005347.s001.tif]

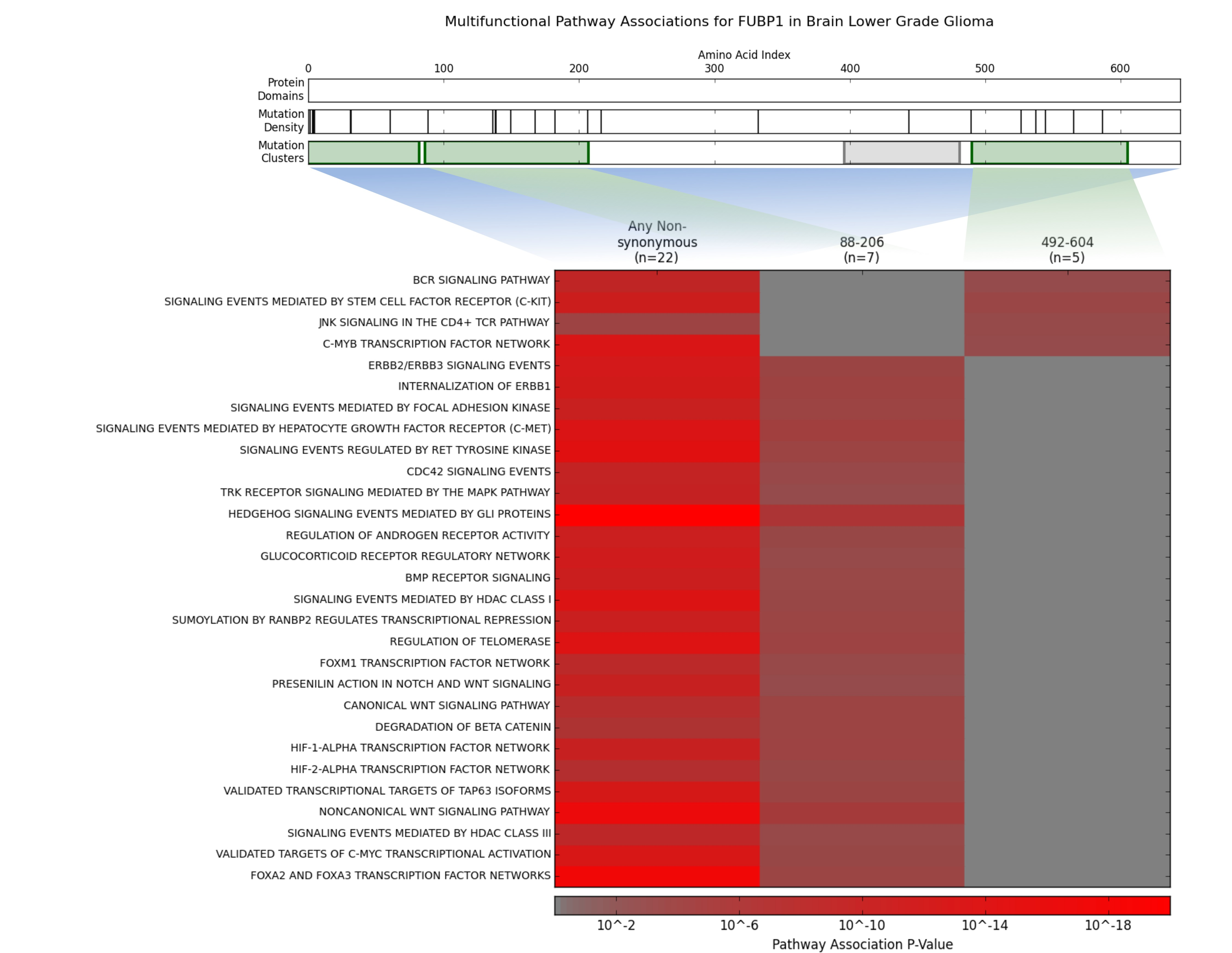

Supplement: S2 Fig — Clusters without significant pathway associations are omitted for clarity. A FDR of 1% was used to filter for significance. (TIF) [file pcbi.1005347.s002.tif]

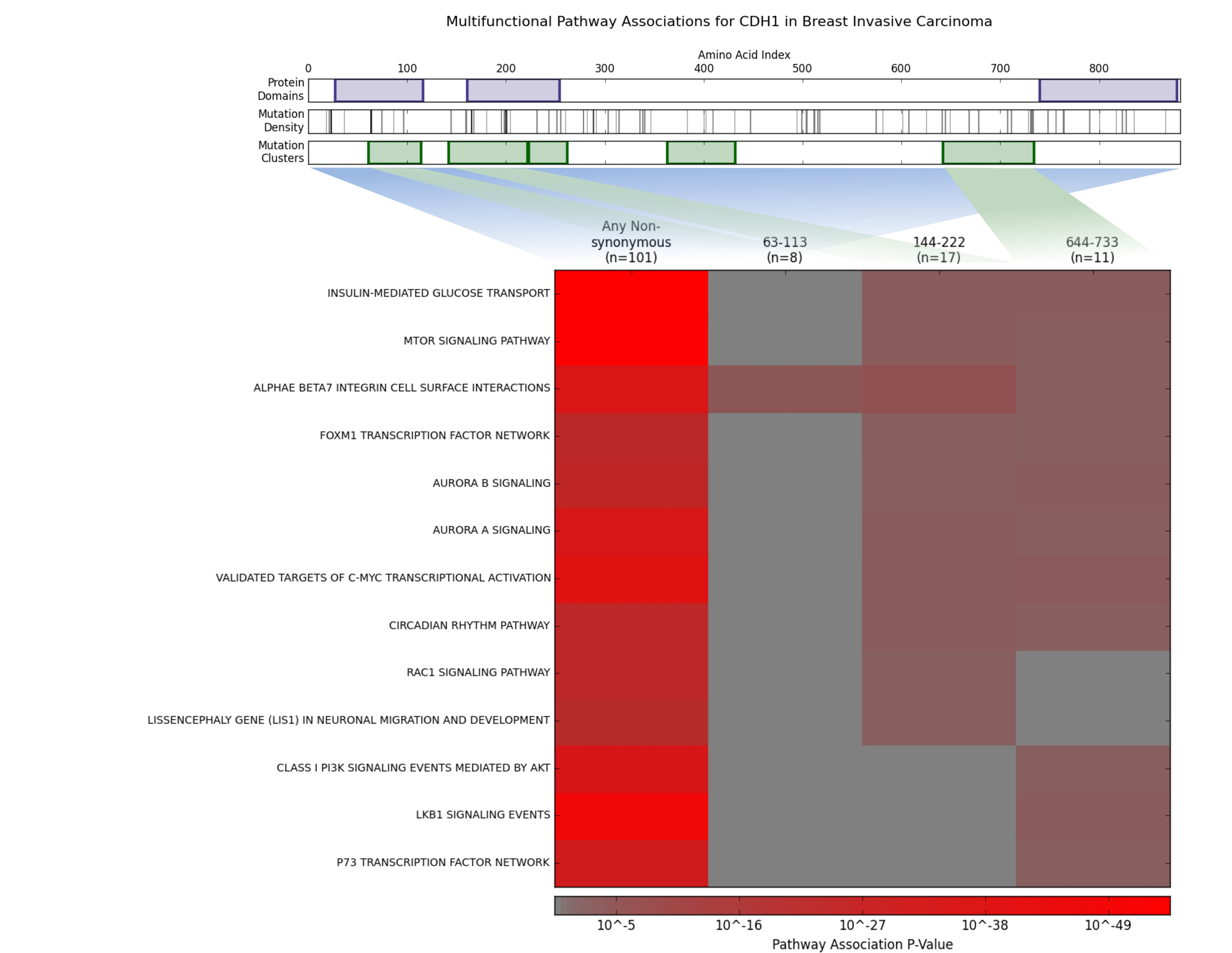

Supplement: S3 Fig — Clusters without significant pathway associations are omitted for clarity. A FDR of 1% was used to filter for significance. (TIF) [file pcbi.1005347.s003.tif]

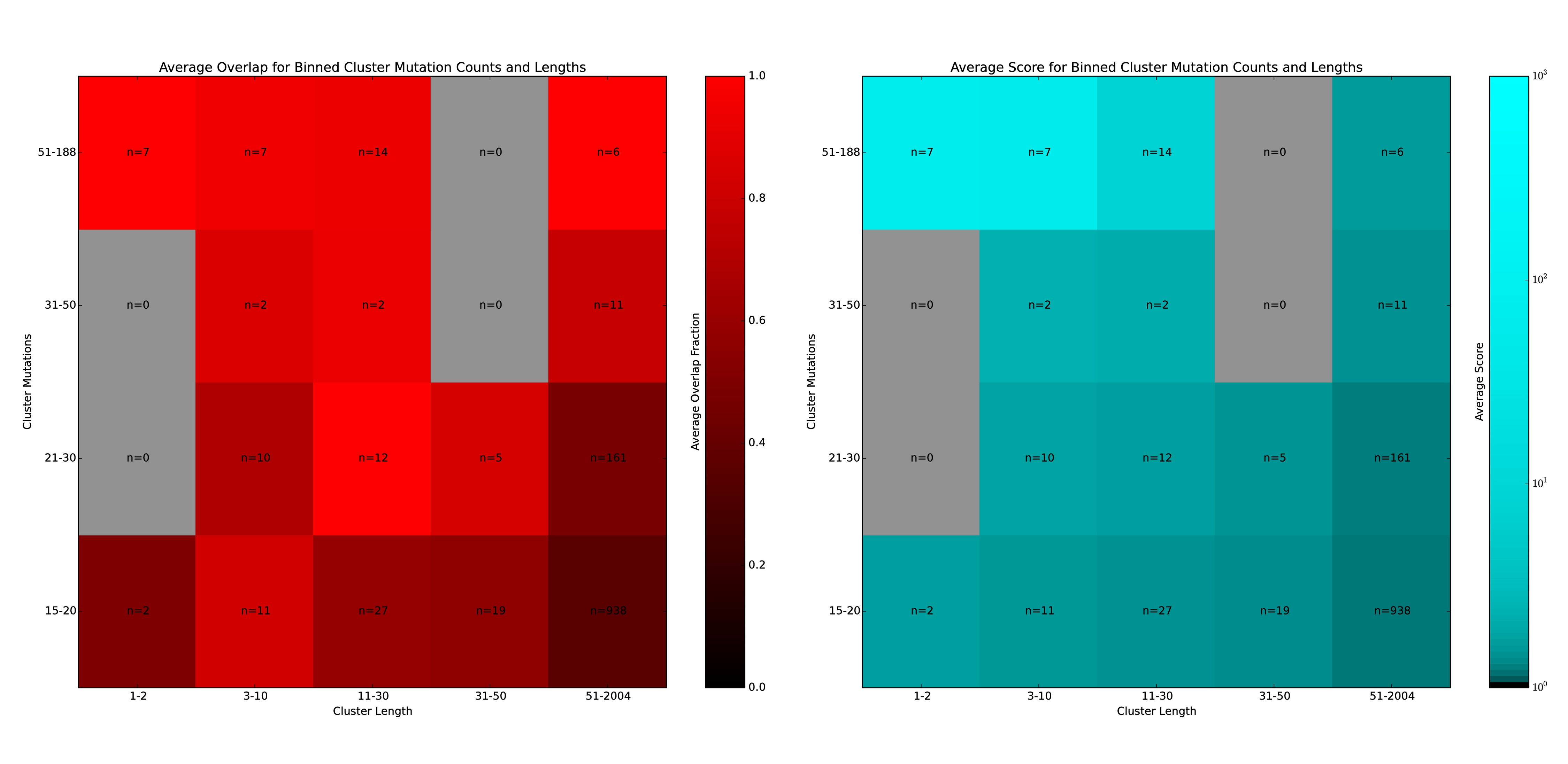

Supplement: S4 Fig — Left) Average overlap percentage for clusters from two partitions (binned by length and mutation count). Gray boxes contain no items. Number of clusters in each bin is indicated by n. Right) Average cluster score for the same binned clusters showing that this score is a reasonable proxy for robustness. (TIF) [file pcbi.1005347.s004.tif]

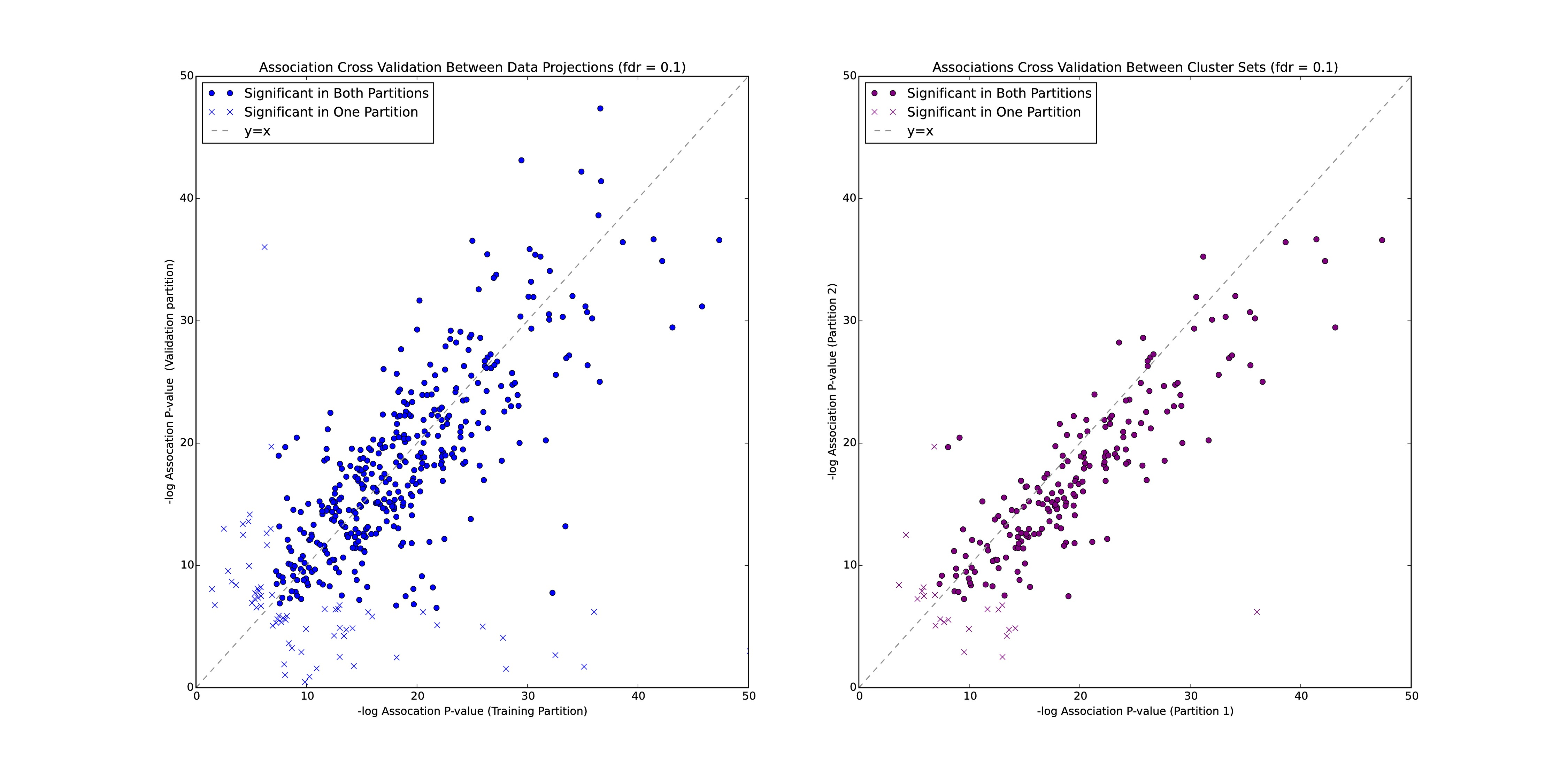

Supplement: S5 Fig — Left) This plot shows association robustness. Data was separated into two partitions A and B. Data from A was used to generate the clusters (“training partition”). Data from B (the validation partition) is compared to A by projecting each partition separately onto the same set of clusters and comparing the pathway associations. This process was then repeated with using B as the training partition and A as the validation partition on a different set of clusters. Right) This plot shows M2C plus association robustness. Here, partition A and partition B were both used to generate separate sets of clusters and the downstream association analysis was performed independently. Cluster associations are matched if the one of the two clusters (from partition A and B respectively) overlap the other by at least 50%. (TIF) [file pcbi.1005347.s005.tif]
