# Supplementary material for: Multiscale mutation clustering algorithm identifies pan-cancer mutational clusters associated with pathway-level changes in gene expression
Source: PLoS Comput Biol. 2017 Feb 7;13(2):e1005347. doi: 10.1371/journal.pcbi.1005347 (PMC5321471; doi:10.1371/journal.pcbi.1005347)
Supplement: S1 Table Descriptions — This document contains descriptions of the supplemental tables, including specific break downs of what information is in each table and how it is formatted. The actual data can be found in S1 Tables as a single excel spreadsheet or as individual TSV’s from http://m2c.systemsbiology.net/. (DOCX) [file pcbi.1005347.s007.docx]

**Table Descriptions**

This document contains descriptions of the supplemental tables, including specific break downs of what information is in each table and how it is formatted. The actual data can be found in S6 Tables as a single excel spreadsheet or as individual TSV’s from <http://m2c.systemsbiology.net/>.

**Table 1:** TCGA Tumor Type Identifiers

| **Cancer** | **TCGA Cancer Acronym** |
| --- | --- |
| Lung squamous cell carcinoma | LUSC |
| Uterine Carcinosarcoma | UCS |
| Prostate adenocarcinoma | PRAD |
| Kidney renal papillary cell carcinoma | KIRP |
| Rectum adenocarcinoma | READ |
| Liver hepatocellular carcinoma | LIHC |
| Lung adenocarcinoma | LUAD |
| Bladder Urothelial Carcinoma | BLCA |
| Cervical squamous cell carcinoma and endocervical adenocarcinoma | CESC |
| Kidney Chromophobe | KICH |
| Stomach adenocarcinoma | STAD |
| Colon adenocarcinoma | COAD |
| Head and Neck squamous cell carcinoma | HNSC |
| Glioblastoma multiforme | GBM |
| Ovarian serous cystadenocarcinoma | OV |
| Kidney renal clear cell carcinoma | KIRC |
| Breast invasive carcinoma | BRCA |
| Uterine Corpus Endometrial Carcinoma | UCEC |
| Adrenocortical carcinoma | ACC |
| Brain Lower Grade Glioma | LGG |
| Skin Cutaneous Melanoma | SKCM |
| Thyroid carcinoma | THCA |
| Acute Myeloid Leukemia | LAML |

The following section describes the supplemental tables which are included as an external excel file (with different tables as different pages) or as external TSV’s from our Github. Note that the descriptions below have been transposed compared to the tables in the supplement. This means the rows described below are actually columns.

**Table 2 Description:** Lists all the cluster definition outputs from M^2^C, the number of mutations in each cluster, the cluster score, and any overlapping protein domains from PFAM.

| **Column Number** | **Column Name** | **Description** |
| --- | --- | --- |
| 1 | Gene | Gene Code |
| 2 | cluster | Start, Stop |
| 3 | Cluster score | $\log\frac{Emission probability by gaussian cluster}{Emission probability under null hypothesis}$ |
| 4 | Number of mutations | # |
| 5 | Gaussian weight | Weight of the corresponding Gaussian from the mixture model |
| 6 | Gaussian Mean | Mean of the corresponding Gaussian from the mixture model |
| 7 | Gaussian Standard Deviation | Standard Deviation of the corresponding Gaussian from the mixture model |
| 8 | Overlapping protein domains | PFam Domain Identifier |

**Table 3 Description:** Lists all the binary cluster assignments to specific tumor types.

| **Column Number** | **Column Name** | **Column Description** |
| --- | --- | --- |
| 1 | cancer | TCGA Cancer (tumor type) Code |
| 2 | Gene | Gene Code |
| 3 | Cluster | Start, Stop |
| 4 | Missense mutations | # |
| 5 | Nonsense mutations | # |
| 6 | Synonymous mutations | # |
| 7 | Included in GEXP analysis | Yes/no [yes if >4 non-synonymous mutations in the cluster |

**Table 4 Description:** Detailed statistics characterizing each tumor type by the clusters inside of it.

| **Column number** | **Column name** | **Column description** |
| --- | --- | --- |
| 1 | Cancer | Full name |
| 2 | TCGA Cancer Acronym | 4-letter code |
| 3 | Total Clusters | Percent of all mutations which are non-synonymous |
| 4 | Genes with clusters | # |
| 5 | Average clusters / gene | # |
| 6 | Average cluster length | (amino acid units) |
| 7 | Combined length of all genes with clusters | (amino acid units) |
| 8 | Combined length of all clusters | (amino acid units) |
| 9 | Fraction of genes covered by clusters | (amino acid units) |
| 10 | Total mutations in genes with clusters | # |
| 11 | Fraction of mutations inside clusters | # |
| 12 | Total non-synonymous mutations in genes with clusters | # |
| 13 | Fraction of non-synonymous mutations in genes with clusters | # |
| 14 | Fraction of mutations in clusters / fractions of genes covered by clusters | # |
| 15 | Fraction of non-synonymous mutations in clusters / fractions of genes covered by clusters | # |

**Table 5 Description:** Cancer type mutation cluster enrichment analysis

| **Column Number** | **Column Name** | **Column Description** |
| --- | --- | --- |
| 1 | Cancer | TCGA Code |
| 2 | Gene | Gene Code |
| 3 | Cluster | Start, Stop |
| 4 | Enrichment P-value | From Fisher’s Exact Test |
| 5 | Odds | Computed by Scipy |
| 6 | False Discovery Rate | .01, .05, .1, or .25 |
| 7 | # Samples containing mutations in the cluster and of the same tumor-type | # |
| 8 | # Samples containing mutations in the cluster and not of the same tumor-type | # |
| 9 | # Samples not containing mutations in the cluster and of the same tumor-type | # |
| 10 | # Samples not containing mutations in the cluster and not of the same tumor-type | # |

**Table 6A Description:** Significant associations between clusters (and any non-synonymous features) and global changes in gene expression.

| **Column Number** | **Column Name** | **Column Description** |
| --- | --- | --- |
| 1 | gene | Gene code |
| 2 | cluster | Start, Stop |
| 3 | Cancer | TCGA cancer code |
| 4 | Global gene expression association p-value | EBM association P-value with all gene expression data |
| 5 | False discovery rate | False discovery threshold used* |
| 6 | Missense mutations | # of missense mutations in the cluster |
| 7 | Nonsense mutation | # of nonsense mutations in the cluster |
| 8 | Synonymous mutations | # of synonymous mutations in the cluster |
| 9 | Non-synonymous less than cluster | TRUE/FALSE^†^ |
| 10 | Cluster less than non-synonymous | TRUE/FALSE^‡^ |
| 11 | Overlapping domains | PFam Domain Identifier |

**Table 6B Description:** Significant associations between oncodriveCLUST clusters and global changes in gene expression.

Same as Table 6A, but only columns 1-10.

**Table 6C Description:** Significant associations between Pfam domains and global changes in gene expression.

Same as Table 6A, but only columns 1-10.

**Table 6D Description:** Associations between non-synonymous mutations not in any cluster and global changes in gene expression. No significance testing was done – results corresponding to significant M^2^C associations are reported if greater than 4 non-synonymous mutations lie outside of any cluster in a particular tumor type.

| **Column Number** | **Column Name** | **Column Description** |
| --- | --- | --- |
| 1 | Cancer | TCGA Code |
| 2 | Gene | Gene Code |
| 4 | P-value | # |

**Table 7A Description:** Significant associations between clusters (and any non-synonymous features) and pathway level changes in gene expression

| **Column Number** | **Column Name** | **Column Description** |
| --- | --- | --- |
| 1 | gene | Gene code |
| 2 | cluster | Start, Stop |
| 3 | Cancer | TCGA cancer code |
| 4 | pathway | Pathway name from the Pathway Interaction Database |
| 5 | Global gene expression association p-value | EBM association P-value with all gene expression data |
| 6 | False discovery rate | False discovery threshold used* |
| 7 | Missense mutations | # of missense mutations in the cluster |
| 8 | Nonsense mutation | # of nonsense mutations in the cluster |
| 9 | Synonymous mutations | # of synonymous mutations in the cluster |
| 10 | Non-synonymous less than cluster | TRUE/FALSE^†^ |
| 11 | Cluster less than non-synonymous | TRUE/FALSE^‡^ |
| 12 | Overlapping domains | PFam Domain Identifier |
| 13 | Pathway Size | # of genes in the pathway |
| 14 | count of significantly increased gene expression features | # of upregulated gene expression features |
| 15 | count of significantly decreased gene expression features | # of downregulated gene expression features |
| 16 | list of significantly increased gene expression features | gene1, p-val; gene2, p-val; … |
| 17 | list of significantly decreased gene expression features | gene1, p-val; gene2, p-val; … |

*Threshold of 0.01, 0.05, 0.1, .25. Computed using the Benjamini–Hochberg method.

†Is the association P-value between a non-synonymous mutation in gene X and the gene expression features less than the corresponding P-values for all clusters in X.

‡Is the association P-value between a given cluster in gene X and the gene expression feature less than the corresponding any non-synonymous feature P-value.

**Table 7B Description:** Significant associations between oncodriveCLUST clusters and pathway level changes in gene expression

Same as Table 6A, but only columns 1-11.

| **Gene Expression Association Overview** | | | | | | | | | |
| --- | --- | --- | --- | --- | --- | --- | --- | --- | --- |
| **M^2^C** |  | **Global GEXP Associations** | | | | **Pathway GEXP Associations** | | | |
|  | ***False Discover Rate*** | ***1%*** | ***5%*** | ***10%*** | ***25%*** | ***1%*** | ***5%*** | ***10%*** | ***25%*** |
|  | **Mutation Clusters with Sig. Associations** | 24 | 33 | 44 | 86 | 51 | 98 | 148 | 246 |
|  | **Any Non-Synonymous with Sig. Associations** | 20 | 42 | 61 | 112 | 76 | 139 | 175 | 223 |
|  | **Total Mutation Cluster Sig. Associations** | 30 | 40 | 55 | 105 | 2200 | 3398 | 4545 | 8193 |
|  | **Total Any Non-Synonymous Sig. Associations** | 32 | 59 | 82 | 150 | 3772 | 5319 | 6762 | 11779 |
|  | **Associations with**  **P_Cluster_ < P_Any Non-Synonymous_** | 9 | 13 | 19 | 35 | 643 | 875 | 1096 | 2404 |
| **OncodriveCLUST** |  | **Global GEXP Associations** | | | | **Pathway GEXP Associations** | | | |
|  | ***False Discover Rate*** | ***1%*** | ***5%*** | ***10%*** | ***25%*** | ***1%*** | ***5%*** | ***10%*** | ***25%*** |
|  | **Clusters with Sig. Associations** | 22 | 33 | 35 | 48 | 41 | 59 | 76 | 122 |
|  | **Total Cluster Sig. Associations** | 28 | 45 | 51 | 77 | 2341 | 3670 | 4774 | 7802 |
|  | **Associations with**  **P_Cluster_ < P_Any Non-Synonymous_** | 5 | 9 | 9 | 14 | 665 | 892 | 1096 | 1842 |
| **Pfam Domains** |  | **Global GEXP Associations** | | | | **Pathway GEXP Associations** | | | |
|  | ***False Discover Rate*** | ***1%*** | ***5%*** | ***10%*** | ***25%*** | ***1%*** | ***5%*** | ***10%*** | ***25%*** |
|  | **Domains with Sig. Associations** | 14 | 23 | 31 | 71 | 39 | 78 | 112 | 198 |
|  | **Total Domain Sig. Associations** | 22 | 33 | 44 | 92 | 1904 | 2803 | 3687 | 7053 |
|  | **Associations with**  **P_Domain_ < P_Any Non-Synonymous_** | 6 | 13 | 16 | 35 | 416 | 744 | 1152 | 2742 |

**Table 7C Description:** Significant associations between Pfam domains and pathway level changes in gene expression

Same as Table 6A, but only columns 1-11.

**Table 7D Description:** Associations between non-synonymous mutations not in any cluster and pathway changes in gene expression. No significance testing was done – results corresponding to significant M^2^C associations are reported if greater than 4 non-synonymous mutations lie outside of any cluster in a particular tumor type.

| **Column Number** | **Column Name** | **Column Description** |
| --- | --- | --- |
| 1 | Cancer | TCGA Code |
| 2 | Gene | Gene Code |
| 3 | Pathway | Name |
| 4 | P-value | # |

**Table 8:** Overview of significant gene expression associations detected at a global and a pathway level between Any Non-Synonymous Features and Cluster Features.

*The top two rows show the total number of features (e.g. cluster features or any non-synonymous features) with significant associations. The next four rows count all the significant associations which means a feature can appear multiple times if it is associated in multiple cancers and/or pathways. The next two rows show association counts where the P-value from either a cluster or any non-synonymous feature is less than the other kind of feature from the same gene in the same tumor type (and for the same pathway, in the case of pathway associations). This table is replicated three times in order to compare the features derived from: M^2^C, OncodriveCLUST, and Pfam domains.*

**Table 9 Description:** Details the pathways iand their gene membership used in our pathway association analysis.

| **Pathway** | **Genes** |
| --- | --- |
| PID Pathway Name | gene1;gene2;gene3; |

**Table 10 Description:** Raw TCGA Mutation Data

| **cancer** | **gene** | **tumor sample** | **mutation type** | **amino acid location** | **reference amino acid** | **mutated amino acid** | **protein ID** |
| --- | --- | --- | --- | --- | --- | --- | --- |
| TCGA Code | Gene Code | Sample ID | Description of amino acid change | # | Amino acid of reference sequence | Amino acid in cancer tumor | Protein ID |

**Table 11 Description:** Details cancer cell line mutation calls inside of clusters.

| **Name** | **descr1** | **descr2** | **descr3** | **Cluster 1** | **Cluster 2…** |
| --- | --- | --- | --- | --- | --- |
| Cell Line Name | Cancer Type Identifiers | | | Gene:start-stop | … |

**Table 12 Description:** Cell Line Cluster Mutation Enrichment P-values at FDR of 5%

| **Gene Name** | **Gene length (aa)** | **Total length of clusters** | **# mutations across cell line panel** | **# mutations in clusters** | **P-value (binomial test)** |
| --- | --- | --- | --- | --- | --- |
| Gene Code | # | # | # | # | # |

**Table 13 Description:** IC50 Drug Response Mutation Cluster P-Values at a FDR of 10%.

| **Drug Information** | | **Gene and mutation cluster information** | | | |
| --- | --- | --- | --- | --- | --- |
| Drug Name | Drug Target | Gene | Mutation cluster | Any Non-synonymous Mutation P-value | Mutation Cluster P-Value |
