# Supplementary material for: Multiscale mutation clustering algorithm identifies pan-cancer mutational clusters associated with pathway-level changes in gene expression
Source: PLoS Comput Biol. 2017 Feb 7;13(2):e1005347. doi: 10.1371/journal.pcbi.1005347 (PMC5321471; doi:10.1371/journal.pcbi.1005347)
Supplement: S1 Text — This document contains detailed information on where the data used in this work comes from, data processing steps, and an in-depth description of the M2C algorithm. (DOCX) [file pcbi.1005347.s008.docx]

**Title:** Multiscale Mutation Clustering Algorithm Identifies Pan-Cancer Mutational Clusters Associated with Pathway-Level Changes in Gene Expression

**Short Title:** Multiscale Mutation Clustering in TCGA

**Authors:** William Poole^1†^, Kalle Leinonen^1^, Ilya Shmulevich^1^, Theo Knijnenburg^1*‡^, and Brady Bernard^1*‡^

^1^Institute for Systems Biology, Seattle, WA.

†Currently at California Institute of Technology, Pasadena, CA

*Corresponding Authors

‡Contributed Equally

**S1 Text: Data, Methods, and Algorithm Details.** This document contains 3 sections. (A) details the data sources and how they were processed. (B) Discusses how genes were chosen for this study. (C) details how the M^2^C algorithm works.

**A) Data Sources:**

**Pathway Interaction Database Processing:**

A copy of the National Cancer Institute Pathway Ontology Downloaded in September, 2012, was used to construct a directed graph of pathway relationships. Pathways were labelled as leaves if they had no sub-pathways or children. This set of leaf pathways and the genes they contained within were used to identify pathway cluster associations. This approach was taken because many pathways are highly redundant differing by only a few genes. By only using leaf pathways, we limited this redundancy and thereby unnecessary multiple testing. Pathways and the genes within them are listed in SI Table 9.

**TCGA Firehose:**

Mutation calls and gene expression data were downloaded for call cancer types on 05/17/2015. This data can be downloaded from: http://ezid.cdlib.org/id/doi:10.7908/C1K64H78 or <http://gdac.broadinstitute.org/runs/analyses__2014_10_17/data/>. Filtered raw mutation data is also available in T10 in S1 Tables.

**B) Selecting Genes for Our Clustering Algorithm:**

An initial list of 628 genes was compiled by taking the highest rated genes from Mutsig using a q-value threshold of 0.1. These genes were further filtered to ensure that there are in total at least 10 mutations in each gene added together across all 23 cancer types considered. This resulted in a list of 549 genes which we ran our multiscale clustering algorithm on. The cancer types considered are: ACC, BLCA, BRCA, CESC, COAD, GBM, HNSC, KICH, KIRC, KIRP, LAML, LGG, LIHC, LUAD, LUSC, OV, PRAD, READ, SKCM, STAD, THCA, UCEC, and UCS.

Additionally, we compiled a list of top Mutsig genes by taking the union of the top five genes for each tumor type provides they had a q-value less than or equal to 0.1. This list included many of the most well studied cancer genes.

**C) The Multiscale Information-Based Clustering Algorithm**:

**The Multi-Bandwidth Mixture Model:**

1. **Starting Data:** First, raw TCGA nucleotide mutation calls are converted into amino acid-space for each gene. Each amino acid in a protein is given an index from 1 to_­­_ *L* where *L* is the length of the protein. Amino acid mutations are classified as synonymous, non-synonymous, and nonsense (insertion, deletion, and stop). The raw data used to identify mutation clusters is the combined set of all kinds of mutations from the 23 tumor types listed above. We include synonymous mutations in our data set because our mixture model explicitly included a uniform distribution to represent background noise, which we also believe is reflected by the synonymous mutations in a gene.
2. **Kernel Density Estimates:** In order to identify multiple scales of mutation clusters, we use kernel density estimates at different bandwidths. Specifically we used Gaussian kernels across the following bandwidth (scales) in amino-acid units: 2, 3, 4, 5, 6, 8, 10, 12, 14, 18, 22, 26, 30, 38, 46, 54, 62, 78, 94, 110, 126, 158, 190, 222, 254, 318, 382, and 446. This process created 28 continuous mutation density estimates denoted *K_h_(x)* where *x* is the position along the gene in amino acid-space and $h$ is the bandwidth. This set of bandwidths was chosen because it is broad and thoroughly covers the set of possible scales without being overly redundant. Additionally it ensures the algorithm is robust – minor changes in this set of bandwidths do not significantly change the clusters generated by the algorithm (data not shown).
3. **Seeding the Mixture Models:** For each scale *h* in part two, an initial set *n_h_* of seed Gaussians and one uniform distribution is identified, where *n_h_* is the number of local maxima in the density estimate. The initial weight of the uniform distribution, denoted *U*, is set as the percentage of synonymous mutations in the gene;

$$U=\frac{M_{silent}}{M_{total}}$$

where *M* is the number of mutations in the gene of a given type. The *_­_i^th^* Gaussian in the mixture model is defined by 3 variables, its weight, its mean, and its standard deviation denoted *w_i_, u_i_,* and *𝜎_I_* respectively. More specifically,

$$G_{i}\left( x; w, \mu, \sigma\right)=\frac{w_{i}}{\sqrt{2\pi\sigma_{i}^{2}}}e^{\left( \frac{x-\mu_{i}}{\sqrt{2}\sigma_{i}} \right)^{2}}.$$

The parameters *w_i_, u_i_,* and *𝜎_I_* are initially estimated from each of the kernel density estimates, *K_h_(x)*, found in step two and later optimized with an expectation maximization (EM) algorithm. Let the amino acid location of the *i^th^* local maximum be denoted *(a_i_, K_h_(a_i_))* . This maximum will be bordered by two local minima, *(b_i_, K_h_(b_i_))* and *(b_i+1_, K_h_(b_i+1_))* to the left and right, respectively. In the edge cases where there is no local minima before or after $a_{i}$, the values *(0, K_h_(0))* and *(L, K_h_(L))* are used as local minima depending upon the case, where *L* is the length of the gene in amino acids. Then, the initial parameters of *G_i_* are given by:

$$w_{i}=m_{i}-\frac{U}{n_{h}} \mu_{i}=a_{i} \sigma_{i}=\frac{{b_{i+1}-b}_{i}}{2}.$$

These seed values are then optimized using an EM algorithm resulting in a final set of clusters for each bandwidth. During this process, it is possible for clusters to removed but not created. Thus the final number of clusters will always be less than or equal to *n*.

**Merging Multiscale Clusters**

1. **Creating a Tree of Clusters:** Step 3 of the algorithm produces a large set of clusters across many bandwidths. These clusters are often duplicates and also typically overlap with each other. We wish to find a set of non-overlapping clusters across all the different scales. This is a difficult optimization problem which involves choosing a set of non-overlapping clusters from all the combinatoric possibilities generated by the different mixture models. We simplify this problem by placing all clusters into a binary tree using a hierarchal clustering algorithm. This simplifies the combinatoric optimization problem to that of choosing between (or merging) two clustering options, each represented by a branch in the tree. To do this, we create a pairwise distance matrix between all clusters. First, all clusters with fewer than 15 mutations are excluded. Second all clusters are paired with the uniform distribution from their given mixture model, represented by a single parameter, *U_h_*. Effectively this means each cluster is defined by 4 terms – its weight, mean, standard deviation, and corresponding uniform noise distribution. Third, in order to avoid redundancy within the cluster tree and increase efficiency, clusters that contain the same set of mutations (but possibly small differences in their other parameters) are averaged together into one single cluster. Let there be *c* clusters denoted *{G_1_, G_2_ … G_c_}* which contain the same mutations. These clusters are merged into one new cluster, *G_merged_*, by taking the arithmetic mean of their parameters;

$w_{merged}=\frac{1}{c}\sum_{i} w_{i},\mu_{merged}=\frac{1}{c}\sum_{i} \mu_{i}, \sigma_{merged}=\frac{1}{c}\sum_{i} \sigma_{i}, \mathrm{and}U_{merged}=\frac{1}{c}\sum_{i} U_{i}$.

Once a total of *N* clusters remain, an *N* by *N* pairwise distance matrix is created. The distance metric between two Gaussian clusters *G_i_(x; w_i_, μ_i_, σ_i_)* and *G_j_(x; w_j_, μ_j_, σ_j_)* is given by the unsigned area between the two curves, specifically

$$d\left( G_{i}, G_{j} \right)= \int_{0}^{L} \left| G_{i}\left( x \right)-G_{j}\left( x \right) \right|dx,$$

Where *L* is the length of the gene in amino acids.

This matrix is used by Scipy’s hierarchical clustering [1] algorithm to produce a binary cluster tree (using default parameters). This tree consists of all Gaussian clusters from different bandwidths used to create the distance matrix.

1. **Flattening the Tree:** A final set of non-overlapping clusters is identified by flattening the binary tree. This is done using a recursive greedy algorithm which locally maximizes the Akaike Information Criteria with finite size correction (AICc) between two adjacent nodes in the tree. Let *L* be the likelihood that a given set of *g* Gaussians and one uniform distribution emit set of *M_total_* mutations found a gene. Then,

$$AICc=2k-2\ln L+\frac{2k(k+1)}{M_{total}-k-1}$$

Where *k = 3g+1* and is the number of parameters in the model.

The greedy algorithm recursively works its way up the tree building a set of non-overlapping clusters. Let *T_i_* be a node in the tree with *T_0_* the root of the tree. Each node *T_i_* has a left and a right child, *C_r_* and *C_l_* respectively. A node is a leaf if it has no children. Furthermore, each leaf represents a Gaussian cluster, *G_i_(x; w_i_, μ_i_, σ_i_)* paired with a uniform distribution, and the mutations inside the cluster. Two clusters *i* and *j* are said to intersect if there exists some mutation *m* such that *m ϵ G_i_* and *m ϵ G_i_*. When the algorithm reaches a given node *i*, if the node is a leaf it returns the set of clusters *{G_i_}.* Otherwise, the algorithm enumerates all the possible sets of non-overlapping clusters from the non-overlapping subsets of clusters returned by *C_r_* and *C_l_*, denoted *S_r_* and *S_l_*, respectively. Let this set of sets be called *Ω = {S_1_…S_k_}.* A non-overlapping subset can be defined as

$S=\left\{ G, H \right| G\cap H= \emptyset, G\in S_{r} \mathrm{and} H\in S_{l}\}$.

For each non-overlapping set *S ϵ Ω*, the AICc is calculated. The algorithm then returns the set *S* which minimizes the AICc to the parent node along with the average uniform distribution from the set of Gaussians contained in *S*. Although, in theory, enumerating all the non-overlapping combinations of nodes is computationally intensive, this is made manageable by the algorithm because each set recursively returned by a child node contains no overlaps, dramatically reducing the number of possible combinations in practice. The pseudo code for the flattening algorithm is as follows:

$\mathbf{Flatten(}\boldsymbol{T}_{\boldsymbol{i}}$**)**

$\mathbf{If}\boldsymbol{T}_{\boldsymbol{i}} \mathbf{is a leaf}\boldsymbol{:}$

$\mathbf{Return}\boldsymbol{\{}\boldsymbol{G}_{\boldsymbol{i}}\boldsymbol{\}}$

$\mathbf{Else:}$

$\mathbf{Recursively call flattenning algorithm:}$

$\boldsymbol{S}_{\boldsymbol{r}}\boldsymbol{=}\mathbf{Flatten(}\boldsymbol{C}_{\boldsymbol{r}}$**)**

$\boldsymbol{S}_{\boldsymbol{l}}\boldsymbol{=}\mathbf{Flatten(}\boldsymbol{C}_{\boldsymbol{l}}\boldsymbol{)}$

$\mathbf{Determine all combinations of non-overlapping clusters between}\boldsymbol{S}_{\boldsymbol{r}} \mathbf{and} \boldsymbol{S}_{\boldsymbol{l}}\boldsymbol{:}$

$\mathcal{S=}\left\{ \boldsymbol{S}_{\boldsymbol{1}}\boldsymbol{,}\boldsymbol{S}_{\boldsymbol{2}}\boldsymbol{\ldots}\boldsymbol{S}_{\boldsymbol{k}}\boldsymbol{|} \boldsymbol{S=}\left\{ \boldsymbol{G, H} \right|\boldsymbol{G\cap H=⊘,G\in}\boldsymbol{S}_{\boldsymbol{r}}\boldsymbol{, H\in}\boldsymbol{S}_{\boldsymbol{l}}\boldsymbol{\}} \right\}$

$\mathbf{Determine AICc for each} \boldsymbol{S\in}\mathcal{S}$**.**

$\mathbf{Return} \boldsymbol{S} \mathbf{with minimum AICc and average noise weight for clusters in}\boldsymbol{S}$

Finally, due to the greedy nature of this algorithm occasionally a non-overlapping cluster is missed as the tree is flattened; in other words the when the final set of non-overlapping clusters *S_f_* is returned, there exists some *G_i_* which does not overlap with any cluster in *S_f_* . This is fixed in a final correction step. In a manner akin to the flattening stage, let *Σ={G_1_…G_N_}* be the set of all clusters in the tree. The correction step finds all non-overlapping subsets between *S_f_* and *Σ.* Again, denoting the set of sets as *Ω = {S_1_…S_k_},* with

$S=\left\{ G, H \right| G\cap H= \emptyset, G\in S_{f} and H\in\Sigma\}$.

For each *S_i_* the AICc is calculated and the *S_i_* with the minimum AICc is selected as the final multi-scale clustering. This corrections step was only necessary on 20 out of the 549 genes considered.

**Bibliography:**

1. Eric Jones and Travis Oliphant and Pearu Peterson and others. (2001--). SciPy: Open source scientific tools for Python. Scipy.org. 2016: 9, 30. Available: http://www.scipy.org/
